# Ionic Liquid Enabled High‐Energy‐Density Solid‐State Lithium Batteries with High‐Areal‐Capacity Cathode and Scaffold‐Supported Composite Electrolyte

**DOI:** 10.1002/smll.202503865

**Published:** 2025-07-26

**Authors:** Tzu‐Yu Kuo, Jagabandhu Patra, Cheng‐Chia Chen, Chun‐Chen Yang, Chien‐Nan Hsiao, Tsai‐Fu Chung, Chung‐Jen Tseng, Rajendra S. Dhaka, Chien‐Te Hsieh, Ju Li, Jeng‐Kuei Chang

**Affiliations:** ^1^ Department of Materials Science and Engineering National Yang Ming Chiao Tung University 1001 University Road Hsinchu 30010 Taiwan; ^2^ Hierarchical Green‐Energy Materials (Hi‐GEM) Research Center National Cheng Kung University 1 University Road Tainan 70101 Taiwan; ^3^ Battery Research Center of Green Energy Ming Chi University of Technology 84 Gongzhuan Road New Taipei City 243303 Taiwan; ^4^ National Applied Research Laboratories Taiwan Instrument Research Institute 20 R&D Road, East District Hsinchu 30010 Taiwan; ^5^ Department of Mechanical Engineering National Central University 300 Jhong‐Da Road Taoyuan 320317 Taiwan; ^6^ Department of Physics Indian Institute of Technology Delhi Hauz Khas New Delhi 110016 India; ^7^ Department of Chemical Engineering and Materials Science Yuan Ze University 135 Yuandong Road Taoyuan 32003 Taiwan; ^8^ Department of Nuclear Science and Engineering and Department of Materials Science and Engineering Massachusetts Institute of Technology 77 Massachusetts Avenue Cambridge MA 02139 USA; ^9^ Department of Chemical Engineering Chung Yuan Christian University 200 Chung Pei Road Taoyuan 32023 Taiwan; ^10^ R&D Center for Membrane Technology 200 Chung Pei Road, Chungli District Taoyuan 32023 Taiwan

**Keywords:** composite cathode, high cathode loading, Li‐ion conductor, solid electrolyte, thin electrolyte layer

## Abstract

Solid‐state lithium batteries (SSLBs) with composite solid electrolytes (CSEs) offer enhanced energy density and high safety. However, their performance is hindered by large thickness and limited Li⁺ conductivity of CSEs, and large electrode/electrolyte interface resistance. This study develops an 18 µm‐thick CSE using a polyethylene scaffold, which incorporates garnet‐type Li_6.25_La_3_Zr_2_Ga_0.25_O_12_ (LLZGO) oxide and an ionic liquid (IL) additive in a poly(vinylidene fluoride‐co‐hexafluoropropylene)/polypropylene carbonate matrix, achieving a high ionic conductivity of 8.6 × 10^−4^ S cm^−1^ at 30 °C. The IL increases Li^+^ conduction of the CSE and reduces the interfacial resistance. The constructed LiNi_0.8_Co_0.1_Mn_0.1_O_2_ (NCM‐811)||CSE||Li cell shows remarkable charge–discharge performance. This study also integrates LLZGO and *N*‐propyl‐*N*‐methylpyrrolidinium bis(trifluorosulfonyl)imide (PMP‐TFSI) IL into a thick NCM‐811 cathode. The synergy between LLZGO and PMP‐TFSI within the cathode is examined. A high‐mass‐loading cathode with ≈20 mg cm^−2^ NCM‐811 is developed. The resulting composite cathode||CSE||Li cell achieves an areal capacity of ≈4 mAh cm^−2^. With the proposed scaffold‐supported CSE and thick NCM/LLZGO/IL composite cathode, the projected energy density of the resulting anode‐free pouch cell is ≈420 Wh kg^−1^. This study demonstrates a scalable and effective strategy for fabricating an oxide‐based SSLB with a high energy density and enhanced Li^+^ transport properties.

## Graphical abstract

A flexible, high Li^+^ conduction, scaffold‐supported composite solid electrolyte with a thickness of ≈18 µm and a composite cathode that shows a high areal capacity of ≈4 mAh cm^−2^ are developed. The synergistic effects of Li_6.25_La_3_Zr_2_Ga_0.25_O_12_ and ionic liquid within both the composite solid electrolyte and the high‐mass‐loading cathode are explored. This study proposes a scalable method to realize high‐energy‐density SSLBs.

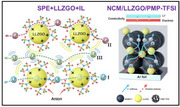

## 1 Introduction

With the growing demand for energy and the need for stable energy supply, research on advanced energy storage devices has become imperative. Among various energy storage devices, lithium batteries (LiBs) stand out due to their high energy density, high power density, long cycle life, and great versatility.^[^ ^1^ ^]^ Improving the safety and energy density of LiBs is crucial for broadening LiB applications, such as electric vehicles and portable electronics. A major safety concern for LiBs is thermal runaway (where the accumulated heat ignites a conventional organic liquid electrolyte),^[^ ^2^, ^3^ ^]^ which can be triggered by physical abuse, overcharging, high temperature, or a short circuit. Traditional liquid electrolytes are highly flammable and degrade over time, especially at elevated temperatures and voltages. Solid‐state electrolytes (SSEs) are less susceptible to such problems,^[^ ^4^ ^]^ potentially increasing the safety and operational lifespan of LiBs. Solid‐state lithium batteries (SSLBs) have thus become a promising solution for future energy storage.^[^ ^5^, ^6^ ^]^ Recently, composite solid electrolytes (CSEs), composed of a polymer, Li salt, and a Li^+^‐conducting inorganic solid electrolyte (ISE), have gained a lot of attention.^[^ ^7^, ^8^ ^]^ CSEs are attractive because they combine the advantages of solid polymer electrolytes (SPEs) and ISEs, providing balanced physicochemical properties.^[^ ^9^ ^]^ There are several types of ISE, including sulfides, halides, oxynitrides, NASICON compounds, perovskite oxides, and garnet oxides.^[^ ^10^, ^11^ ^]^ Garnet‐structure Li_7_La_3_Zr_2_O_12_‐based oxide has adequate Li^+^ conductivity, excellent chemical stability, a wide electrochemical stability window, and low toxicity.^[^ ^12^, ^13^ ^]^ Therefore, a CSE with garnet‐type oxide fillers is considered in the present study. Although promising, CSEs still have many challenges,^[^ ^14^ ^]^ such as insufficient mechanical strength, excessive thickness, limited Li^+^ conductivity, and poor interfacial contact with electrodes, which must be addressed before SSLBs can be widely applied.

The development of high‐energy‐density SSLBs includes two primary approaches. The first approach is decreasing SSE thickness.^[^ ^15^ ^]^ The thickness of the SSE layer influences the energy density of SSLBs, as this layer is essentially an inactive component in the cell. A thick SSE layer not only increases weight but also reduces the space available for loading the electroactive materials, decreasing the cell energy density. Of note, a thin SSE is favorable for Li^+^ conduction, which is beneficial for cell charge–discharge performance. CSEs fabricated via a conventional tape‐casting method typically exceed 50 µm in thickness,^[^ ^16^, ^17^ ^]^ which is unfavorable for practical LiBs. In contrast, thin CSEs often suffer from inevitable internal defects and insufficient mechanical strength, leading to an increased risk of cell short circuits and lithium dendrite penetration.^[^ ^18^ ^]^ To address these issues, innovative designs for thin yet robust CSEs are urgently required. One strategy is to impregnate the CSE into porous scaffolds,^[^ ^19^, ^20^, ^21^ ^]^ which act as mechanical support to firmly separate the anodes and cathodes. This technique has been used to fabricate thin CSEs that maintain reliability and stability. However, scaffold‐supported CSEs still have several difficulties, including unsatisfactory Li^+^ conductivity and poor connection with anodes/cathodes due to their high rigidity.^[^ ^22^ ^]^ Therefore, in the present study, an ionic liquid (IL), characterized by non‐flammability, near‐zero vapor pressure, high decomposition temperature, and intrinsic ionic conductivity,^[^ ^23^, ^24^, ^25^ ^]^ is first incorporated in scaffold‐supported CSEs. The interactions of the IL with the polymer substances and oxide particles are examined in detail.

The second approach for realizing high‐energy‐density SSLBs is increasing cathode thickness.^[^ ^26^ ^]^ A thicker cathode allows for higher active material loading (reducing the proportion of inactive components), which increases the total number of Li^+^ ions that can be stored within a cell. A high energy density is crucial for diverse applications, such as portable devices and electric vehicles, which require substantial energy storage per unit weight or volume. However, SSEs cannot easily infiltrate thick cathodes, leading to high Li^+^ transport resistance and sluggish charge transfer kinetics due to the lack of effective Li^+^ conduction pathways. This deteriorates the charge–discharge performance of SSLBs.^[^ ^27^ ^]^ To overcome these limitations, extra Li^+^ conductors need to be incorporated to establish Li^+^ conduction channels within the thick cathodes.^[^ ^28^ ^]^ A few studies introduced Li^+^‐conducting oxides to prepare composite cathodes for CSE‐based SSLBs. For instance, Kim et al. proposed a composite cathode, where Li_6.25_Al_0.25_La_3_Zr_2_O_12_ was combined with LiNi_0.4_Co_0.2_Mn_0.4_O_2_. The active material loading was 5 mg cm^−2^. They operated the cell at 0.1 C, achieving a discharge capacity of ≈122 mAh g^−1^ and an areal capacity of ≈0.61 mAh cm^−2^ at 70 °C.^[^ ^29^ ^]^ Heo et al. fabricated a composite cathode using La_2_(Ni_0.5_Li_0.5_)O_4_ as the Li^+^ conductor. The loading of LiNi_0.8_Co_0.1_Mn_0.1_O_2_ (NCM‐811) was 5 mg cm^−2^. In testing at 0.1 C, the cell showed a specific capacity of ≈144 mAh g^−1^ and an areal capacity of ≈0.72 mAh cm^−2^ at 70 °C.^[^ ^30^ ^]^ López‐Aranguren et al. prepared a composite cathode using an Li_7_La_3_Zr_2_O_12_ Li^+^ conductor. The cell with an LiNi_0.6_Co_0.2_Mn_0.2_O_2_ composite cathode was tested at 0.05 C, showing a specific capacity of 150 mAh g^−1^ and an areal capacity of 1.6 mAh cm^−2^ at 70 °C.^[^ ^31^ ^]^ Park et al. developed a composite cathode using Li_1.3_Al_0.3_Ti_1.7_(PO_4_)_3_ and LiNi_0.8_Co_0.15_Al_0.05_O_2_ (the mass loading was 2–3 mg cm^−2^). At an operation temperature of 60 °C, the composite cathode had a capacity of 184 mAh g^−1^ at 9 mA g^−1^ and an areal capacity of 0.37–0.55 mAh cm^−2^.^[^ ^32^ ^]^ It is noted that the above cells were tested at elevated temperatures and the cathode areal capacity was not high (the highest value reported for oxide‐based composite cathodes for CSE‐based SSLBs is 1.6 mAh cm^−2^ at 70 °C). A better design for improving the room‐temperature charge–discharge performance and increasing the mass loading (and thus the areal capacity) of composite cathodes is the goal of this work.

**Figure 1:**
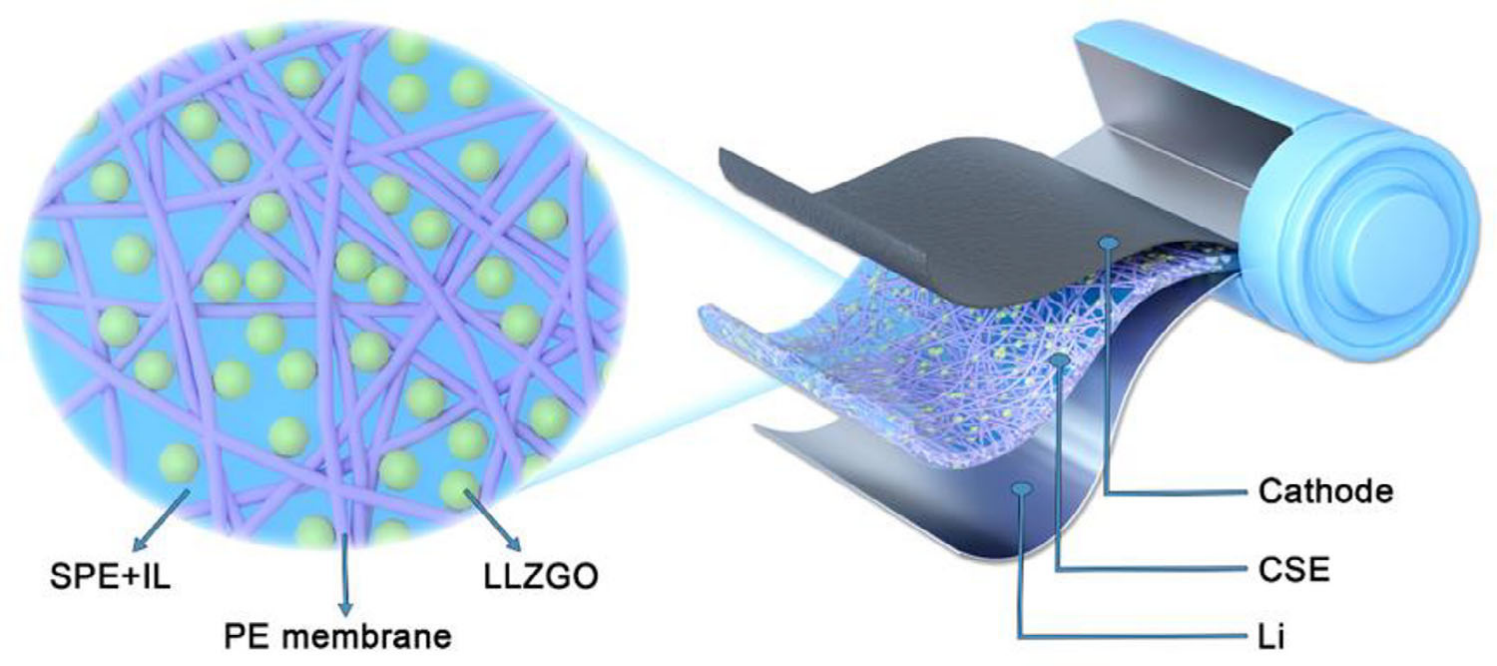
Schematic of scaffold‐supported CSE developed in this study.

In the present study, we use a scaffold support method to develop a thin CSE, which is composed of an SPE, Li_6.25_La_3_Zr_2_Ga_0.25_O_12_ (LLZGO) particles, and an IL additive. Porous polyethylene (PE) membranes with a thickness of ≈16 µm are used as the scaffolds. The SPE is a blend of lithium bis(trifluoromethane)sulfonamide (LiTFSI), polypropylene carbonate (PPC), and poly(vinylidene fluoride‐co‐hexafluoropropylene) (PVDF‐HFP). The IL is composed of 1 M LiTFSI in *N*‐propyl‐*N*‐methylpyrrolidinium bis(fluorosulfonyl)imide (PMP‐FSI) with 1 wt.% lithium difluoro(oxalato)borate (LiDFOB). The PMP‐FSI is selected due to its high ionic conductivity and low viscosity.^[^ ^33^ ^]^ LiDFOB is known to form a stable interfacial layer between the electrolyte and electrode.^[^ ^34^ ^]^ The scheme of the scaffold‐supported CSE is presented in **Figure** **1**. This study attempts to simultaneously incorporate LLZGO particles and IL into a polymer electrolyte for improving both the bulk conductivity and interface impedance of a CSE. The synergistic effects and underlying mechanism of combining LLZGO and IL are investigated. In addition, an LLZGO/IL ionogel is introduced into a high‐mass‐loading cathode to construct continuous Li^+^ conduction pathways. The NCM‐811 active material loading in the developed composite cathode reaches ≈20 mg cm^−2^ (i.e., an areal capacity of ≈4 mAh cm^−2^), which exceeds previously reported values of oxide‐based composite cathodes for CSE‐based SSLBs.^[^ ^35^, ^36^, ^37^, ^38^ ^]^ Furthermore, the projected energy density of the anode‐free pouch cell is 420 Wh kg^−1^, highlighting the competitiveness of the SSE and composite cathode developed in this study, which paves the way toward high‐performance SSLBs.

## 2 Results and Discussion

**Figure 2:**
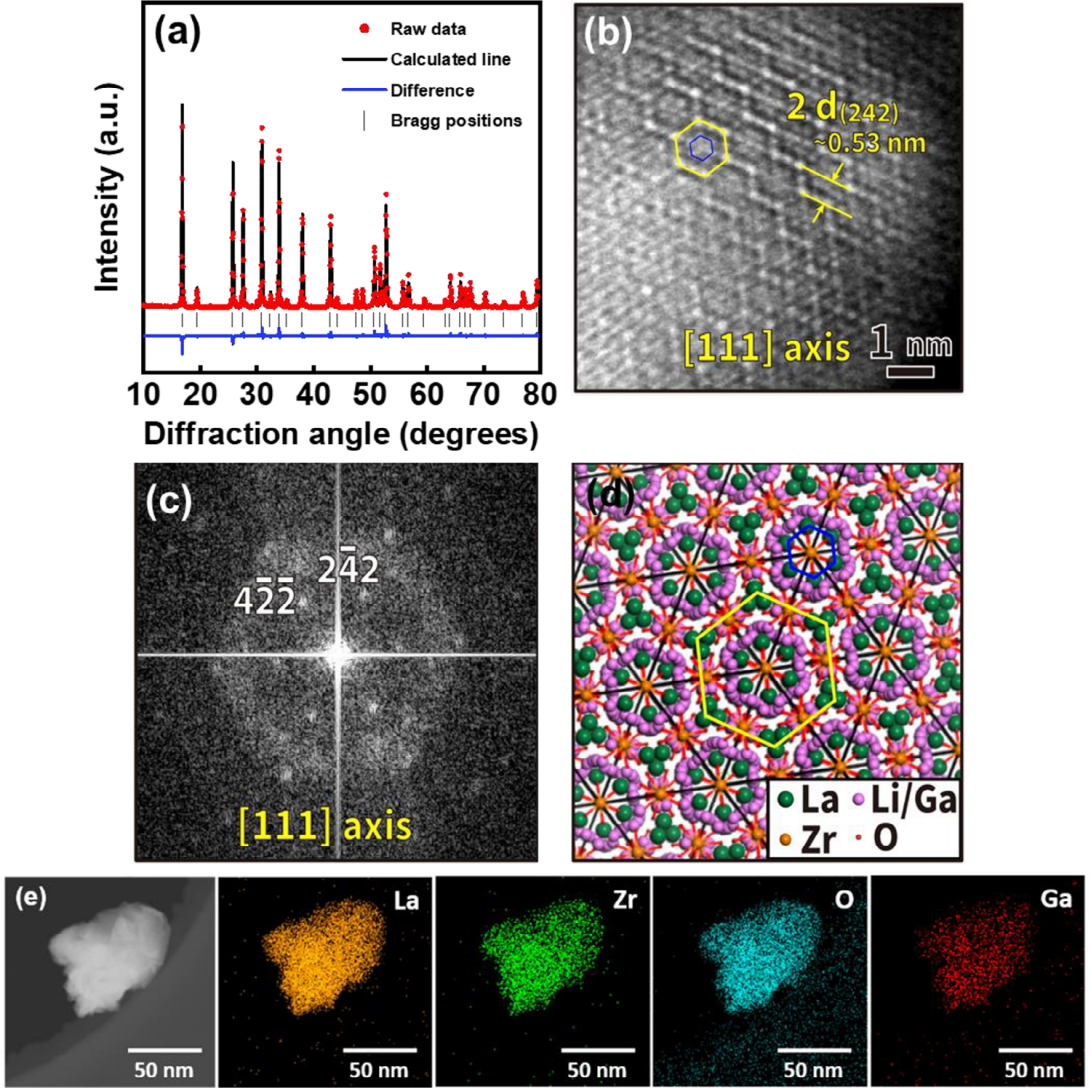
a) XRD pattern and corresponding Rietveld fitting of LLZGO powder. b) HR‐TEM image of LLZGO and c) corresponding FFT diffractogram. d) Calculated structure of LLZGO projected along [111]_LLZGO_ direction. e) EDS elemental mapping data of LLZGO powder.

LLZGO ceramic particles were synthesized via a solid‐state reaction method. The Rietveld refinement of X‐ray diffraction (XRD) data, shown in **Figure** **2**a, indicates that the LLZGO has a cubic garnet structure (PDF 63‐0174).^[^ ^39^ ^]^ The corresponding scanning electron microscopy (SEM) image is shown in Figure S1 (Supporting Information). The size of the LLZGO particles is ≈50 nm. Figure 2b shows a high‐resolution transmission electron microscopy (HR‐TEM) image of the LLZGO, revealing a lattice with a hexagonal atomic arrangement. The atomic configuration includes hexagons of two sizes, indicated by yellow and blue frames, respectively, showing different Z‐contrast along the [111]_LLZGO_ zone axis. The 2*d* spacing of 0.53 nm corresponds to (242)_LLZGO_. Figure 2c shows the corresponding fast Fourier transform diffractogram, confirming the cubic phase of the LLZGO. In Figure 2d, the calculated structure of the LLZGO, which is drawn using the lattice parameters^[^ ^40^, ^41^ ^]^ obtained from a first‐principles calculation, is presented.^[^ ^42^, ^43^ ^]^ For the larger hexagon (indicated by a yellow frame), the different Z‐contrast of the atomic columns along the edges is associated with three‐fold La and Li/Ga‐Zr‐Li/Ga atomic arrangements. For the smaller hexagon (indicated by a blue frame), the uniform Z‐contrast of the atomic columns along the edges is ascribed to the La atomic columns. The energy‐dispersive X‐ray spectroscopy (EDS) mapping results in Figure 2e show a homogenous distribution of all elements within the LLZGO particle.

**Figure 3:**
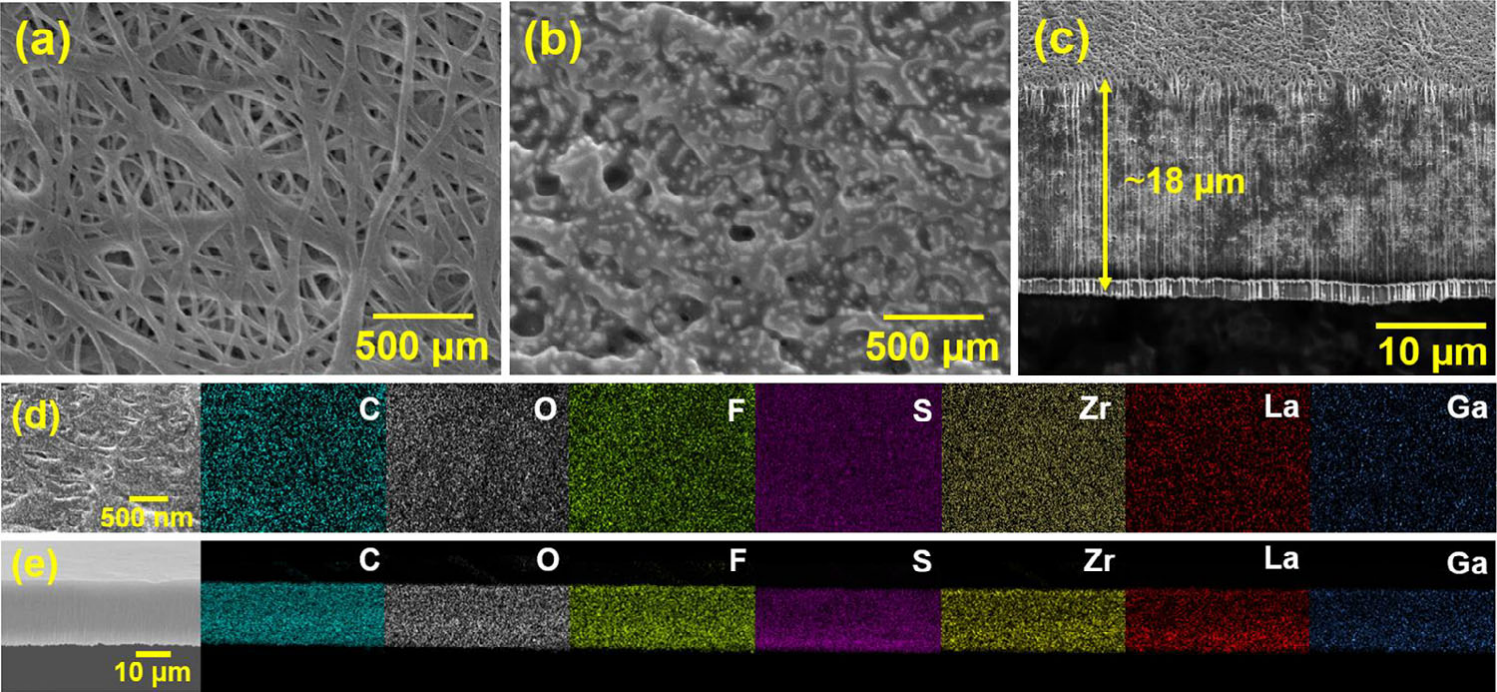
SEM images of PE membranes a) before and b) after SPE+LLZGO+IL infiltration. c) FIB/SEM cross‐section image of PE‐scaffold‐supported CSE. d) Top‐view and e) cross‐section EDS mapping data of PE‐scaffold‐supported CSE.

**Figure** **3**a,b show SEM images of the PE membrane before and after SPE+LLZGO+IL infiltration, respectively. After the incorporation of the SPE, LLZGO, and IL into the PE scaffold, the PE membrane becomes filled, resulting in reduced porosity. Signals corresponding to LLZGO appear in the XRD pattern of the SPE+LLZGO+IL layer, as shown in Figure S2 (Supporting Information), confirming the successful integration of LLZGO into the CSE. A focused ion beam (FIB) was employed to examine the cross‐section morphology of the scaffold‐supported CSE. The FIB/SEM image in Figure 3c shows that the thickness of the CSE was ≈18 µm, which is slightly higher than that (≈16 µm) of the pristine PE. Figure 3d,e respectively show the top‐view and cross‐section EDS mapping data of the CSE layer. Notably, the CSE consists of a homogeneous dispersion of the polymer phase, LLZGO particles, and IL, resulting in a uniform distribution of C, F, La, Zr, Ga, O, and S elements. Figure S3 (Supporting Information) shows the optical appearance of the CSE layer, revealing its high flexibility and robustness. The incorporated IL acts as both a plasticizer in the polymer matrix and a connection layer surrounding the LLZGO particles. As shown in Figure S3 (Supporting Information), no liquid phase appeared on the CSE. The softness and adhesiveness of the CSE layer help address the interfacial problem with the electrodes. To evaluate the mechanical properties of the prepared CSE, tensile testing was conducted. As shown in Figure S4 (Supporting Information), the mechanical strength of the proposed CSE is significantly higher than that of its free‐standing counterpart, which has the same composition except for the absence of the PE scaffold. These results reveal that the PE‐scaffold‐supported method is effective in reinforcing the structure of the solid electrolyte layer.

**Figure 4:**
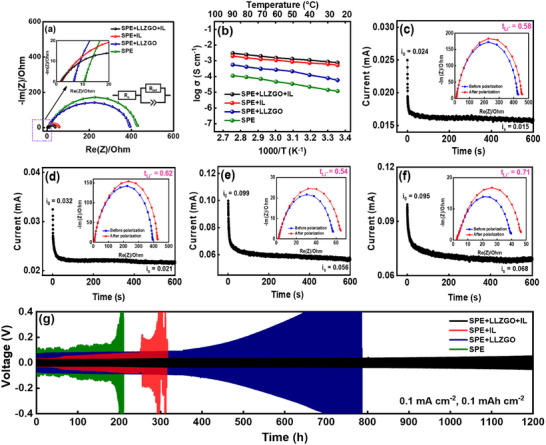
a) EIS spectra and b) ionic conductivity of various SSEs at different temperatures. The *t*_Li_^+^ value measurements of c) SPE, d) SPE+LLZGO, e) SPE+IL, and f) SPE+LLZGO+IL electrolytes. Chronoamperometry curve of various cells measured under 10 mV polarization. *Io* and *Is* represent initial current and steady‐state current, respectively. The insets are the EIS spectra before (blue) and after (red) polarization. g) Lithium plating and stripping curves of various Li||SSE||Li symmetric cells.

To clarify the functions of the developed CSE, we studied four kinds of PE‐scaffold SSE membrane, namely SPE, SPE+LLZGO, SPE+IL, and SPE+LLZGO+IL. **Figure** **4**a shows the electrochemical impedance spectroscopy (EIS) spectra of various Li||SSE||Li cells. The Nyquist spectra can be characterized by the equivalent circuit shown in the figure inset, where *R*_e_ and *R*_int_ represent the electrolyte resistance and electrode/electrolyte interfacial charge‐transfer resistance, respectively.^[^ ^44^ ^]^ The *R*_e_ and *R*_int_ values derived from the EIS spectra at 30 °C are listed in **Table** **1**. As shown, the IL outperforms LLZGO in reducing *R*_e_ and *R*_int_ because it is more ion‐conductive and more effective (as a plasticizer) in decreasing the crystallinity (and thus rigidity) of the SPE,^[^ ^45^ ^]^ resulting in improved interface connection. The SPE+LLZGO+IL CSE shows the optimal properties due to the synergy between LLZGO particles and the IL (the mechanism is discussed later). The Li^+^ mobility in the electrolyte is governed by temperature‐dependent kinetics. Figure 4b shows the ionic conductivities of the four SSEs at various temperatures. At 30 °C, the electrolyte conductivity values for SPE, SPE+LLZGO, SPE+IL, and SPE+LLZGO+IL are 1.4 × 10^−5^, 8.1 × 10^−5^, 6.2 × 10^−4^, and 8.6 × 10^−4^ S cm^−1^, respectively. As shown, the conductivity increases with increasing temperature. The corresponding activation energies of the four SSEs, derived from the Arrhenius equation, are calculated in Figure S5 (Supporting Information). The obtained activation energy values are 0.32 eV for SPE, 0.31 eV for SPE+LLZGO, 0.23 eV for SPE+IL, and 0.19 eV for SPE+LLZGO+IL. The reduced activation energy observed for SPE+LLZGO+IL suggests improved ion transport kinetics,^[^ ^46^ ^]^ potentially enhancing the cell charge–discharge properties, which are investigated later. The latter two samples were further subjected to differential scanning calorimetry (DSC) measurements, and the results are shown in Figure S6 (Supporting Information). The endothermic peak at ≈140 °C is associated with the melting process of the polymer phase.^[^ ^47^ ^]^ It is evident that the peak area, corresponding to the melting enthalpy, of SPE+LLZGO+IL is clearly smaller than that of SPE+IL. This indicates that the crystallinity of the polymer phase in SPE+LLZGO+IL is significantly lower,^[^ ^48^ ^]^ likely due to the combined incorporation of the LLZGO and IL, which disrupts the regular packing of polymer chains. The increased flexibility (i.e., reduced ordering) of polymer chain segments is a key factor contributing to the high Li^+^ conductivity of the SPE+LLZGO+IL layer.^[^ ^49^ ^]^

**Table 1:** Electrolyte resistance (*R*_e_) and electrode/electrolyte interfacial charge‐transfer resistance (*R*_int_) of various Li||SSE||Li cells derived from EIS data.

	SPE	SPE+LLZGO	SPE+IL	SPE+LLZGO+IL
*R*_e_ (Ω)	11	7	2	1
*R*_int_ (Ω)	420	388	55	37

The lithium‐ion transference number (*t*_Li_^+^) is a key parameter of SSEs. The *t*_Li_^+^ values were calculated using the Bruce‐Vincent method.^[^ ^50^ ^]^ The details can be found in the Supporting Information. Figure 4c–f shows the chronoamperometry profiles and the EIS data measured before and after polarization for various SSEs. The *t*_Li_^+^ values obtained are 0.58 for SPE, 0.62 for SPE+LLZGO, 0.54 for SPE+IL, and 0.71 for SPE+LLZGO+IL. It is evident that the addition of LLZGO into the SPE clearly increases *t*_Li_^+^. This is attributed to LLZGO intrinsically having a *t*_Li_^+^ value of 1, selectively transporting Li^+^ ions while suppressing anion mobility.^[^ ^51^ ^]^ In contrast, solely adding IL decreases *t*_Li+_ due to the promoted formation of ion pairs and aggregates.^[^ ^50^ ^]^ Of note, SPE+LLZGO+IL exhibits the highest *t*_Li_^+^ value. Figure S7 (Supporting Information) shows a zeta potential of 49 mV for the LLZGO particles dispersed in PMP‐FSI IL, confirming the presence of positive charge on the LLZGO surface. This positive charge can attract electrolyte anions, promoting ion pair/aggregate dissociation and allowing Li^+^ ions to freely migrate.^[^ ^52^ ^]^ As a consequence, both the overall ionic conductivity and *t*_Li_^+^ increase.

To evaluate the long‐term stability of the SSEs, the lithium plating and stripping of Li||SSE||Li symmetric cells were repeatedly conducted at a current density of 0.1 mA cm^−2^. The plating and stripping reactions were alternated every 1 h. As shown in Figure 4g, SPE exhibits high polarization and a clear short circuit after ≈200 h. SPE+LLZGO is stable in the initial ≈350 h and experiences a gradual polarization increase afterward until cell failure at ≈790 h. Although SPE+IL shows reduced cell polarization, it fails after ≈250 h. Due to the low *t*_Li_^+^ of SPE+IL, anions in the electrolyte easily move, leading to uneven Li deposition.^[^ ^53^ ^]^ Since the growth of Li dendrites cannot be effectively mitigated by the relatively weak electrolyte layer (because there are no LLZGO particles), early failure was observed. In contrast, SPE+LLZGO+IL shows a steady plating/stripping overpotential of ≈25 mV up to 1200 h. This enhanced stability is due to the LLZGO particles acting as ion redistributors^[^ ^54^ ^]^ and having synergistic effects with the IL, producing effective Li^+^ transport pathways across the SSE while maintaining mechanical strength. To validate this hypothesis, the cells after 200 plating/stripping cycles were opened in a dry room (with a dew point of ≈ –50 °C). The optical and SEM images in Figure S8a (Supporting Information) reveal a nonuniform dead Li layer left on the SPE+IL membrane. In contrast, there is almost no Li accumulation on the SPE+LLZGO+IL layer, as shown in Figure S8b (Supporting Information). The higher *t*_Li_^+^ of the latter electrolyte facilitates more uniform Li deposition,^[^ ^55^, ^56^ ^]^ leading to a longer cell lifespan.

**Figure 5:**
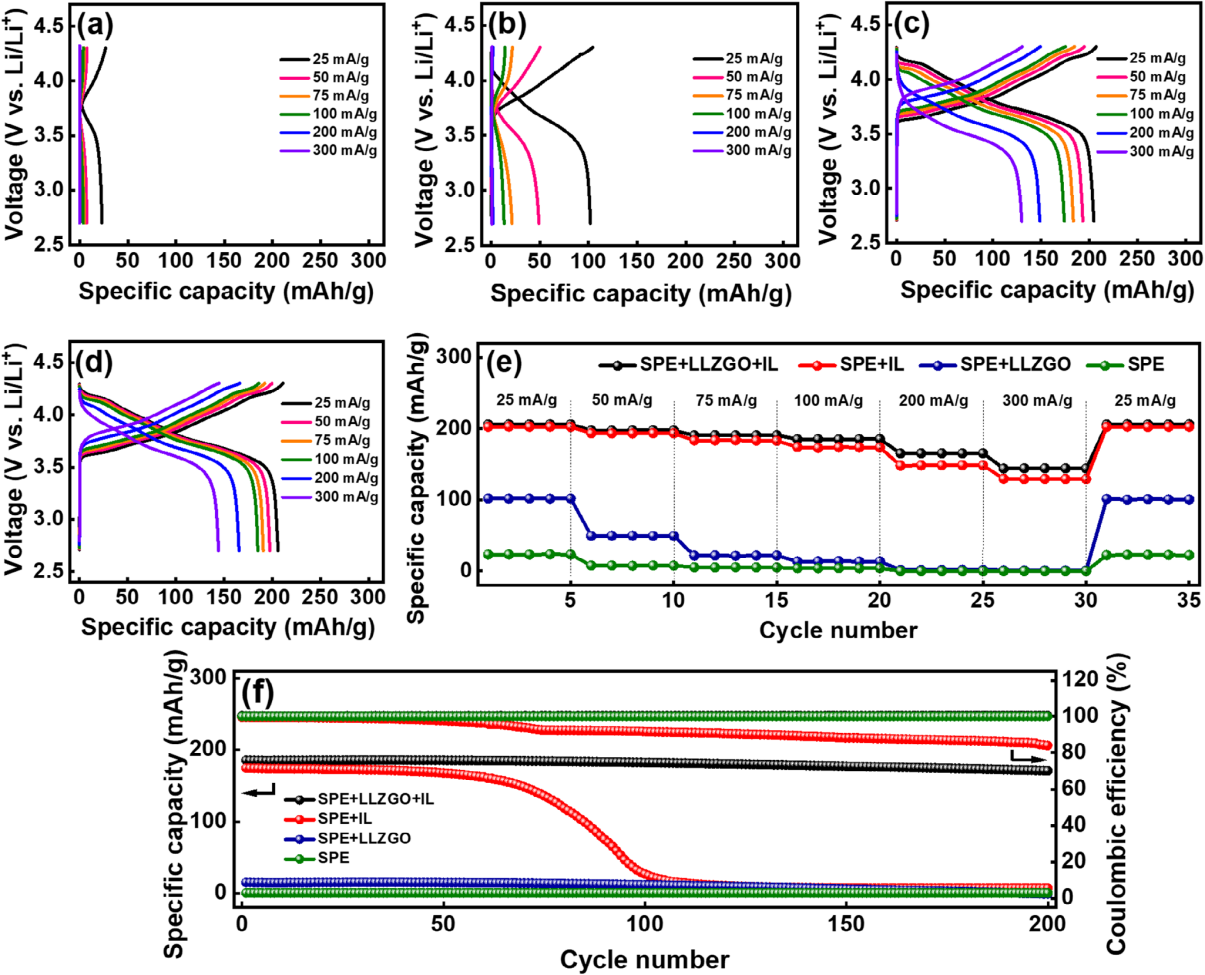
Galvanostatic charge–discharge curves of NCM‐811||Li cells with a) SPE, b) SPE+LLZGO, c) SPE+IL, and d) SPE+LLZGO+IL electrolytes. e) Comparative rate performances and f) cycling stability measured at 100 mA g^−1^ for various NCM‐811||SSE||Li cells.

To evaluate the electrochemical performance of various SSEs, NCM‐811||SSE||Li cells were assembled and tested at 30 °C (the cathode mass loading was ≈2.5 mg cm^−2^). The charge–discharge profiles at various current rates are shown in **Figure** **5**a–d, and the reversible capacities are summarized in **Table** **2**. Figure 5a shows the rate performance of the NCM‐811||SPE||Li cell, which has a low capacity of 23 mAh g^−1^ even at a low current rate of 25 mA g^−1^. The limited ionic conductivity and high interfacial resistance of the SPE restrict cell performance, making the capacities negligible as the current rate increases. Although the NCM‐811||SPE+LLZGO||Li cell incorporates LLZGO to enhance electrolyte conductivity, the high electrode/electrolyte interfacial resistance limits the cell charge–discharge capability (e.g., 102 mAh g^−1^ at 25 mA g^−1^, as shown in Figure 5b). By addressing the interfacial problems through the incorporation of an IL, the NCM‐811||SPE+IL||Li cell shows clear improvement in the electrochemical properties (Figure 5c). Notably, the NCM‐811||SPE+LLZGO+IL||Li cell shows the best performance among all cell configurations, owing to the synergistic combination of LLZGO and the IL within the SSE (Figure 5d). Figure 5e compares the rate performance of all cells. In general, the specific capacities decrease with increasing current density due to the internal cell resistance and kinetic limitations.^[^ ^57^ ^]^ The cell with SPE+LLZGO+IL exhibits superior rate capability, which is associated with lower *R*_e_ and *R*_int_, as shown in Table 1. At 300 mA g^−1^, a specific capacity of 145 mAh g^−1^ is achieved, corresponding to >70% retention compared to the capacity measured at 25 mA g^−1^. In addition, the effects of the amount of LLZGO and IL in the CSE on the cell charge–discharge performance were investigated. As shown in Figure S9 (Supporting Information), when the amount of LLZGO and IL is reduced, the specific capacities of NCM‐811 decrease, especially at high rates. However, we also found that increasing the LLZGO/IL content increases the fluidity of the CSE. Therefore, proper control of the LLZGO/IL amount is crucial for optimizing the cell performance.

**Table 2:** Reversible capacities of NCM‐811 measured at various rates in various SSE cells.

Current density [mA g^−1^]	Reversible capacity [mAh g^−1^]			
	SPE	SPE+LLZGO	SPE+IL	SPE+LLZGO+IL
25	23	102	204	206
50	7	49	193	197
75	5	21	183	190
100	4	13	173	185
200	0	2	148	165
300	0	1	129	145

The cycling stability of various cells measured at 100 mA g^−1^ is shown in Figure 5f. The capacities of NCM‐811||SPE||Li, NCM‐811||SPE+LLZGO||Li, and NCM‐811||SPE+IL||Li cells decay to almost zero after 120 cycles. In contrast, the NCM‐811||SPE+LLZGO+IL||Li cell shows great stability, retaining 92% of its initial capacity after 200 cycles. The IL plasticizer improves the connection between the SSE and electrodes and enhances the interface compatibility.^[^ ^58^ ^]^ Meanwhile, the dispersed LLZGO particles play a crucial role in regulating Li^+^ flux within the SSE, minimizing the ion concentration gradient that could otherwise lead to easy Li dendrite formation.^[^ ^59^ ^]^ In addition, the LLZGO particles provide mechanical strength for suppressing Li dendrite penetration. Together, these factors contribute to the superior cycling stability of the NCM‐811||SPE+LLZGO+IL||Li cell.

**Figure 6:**
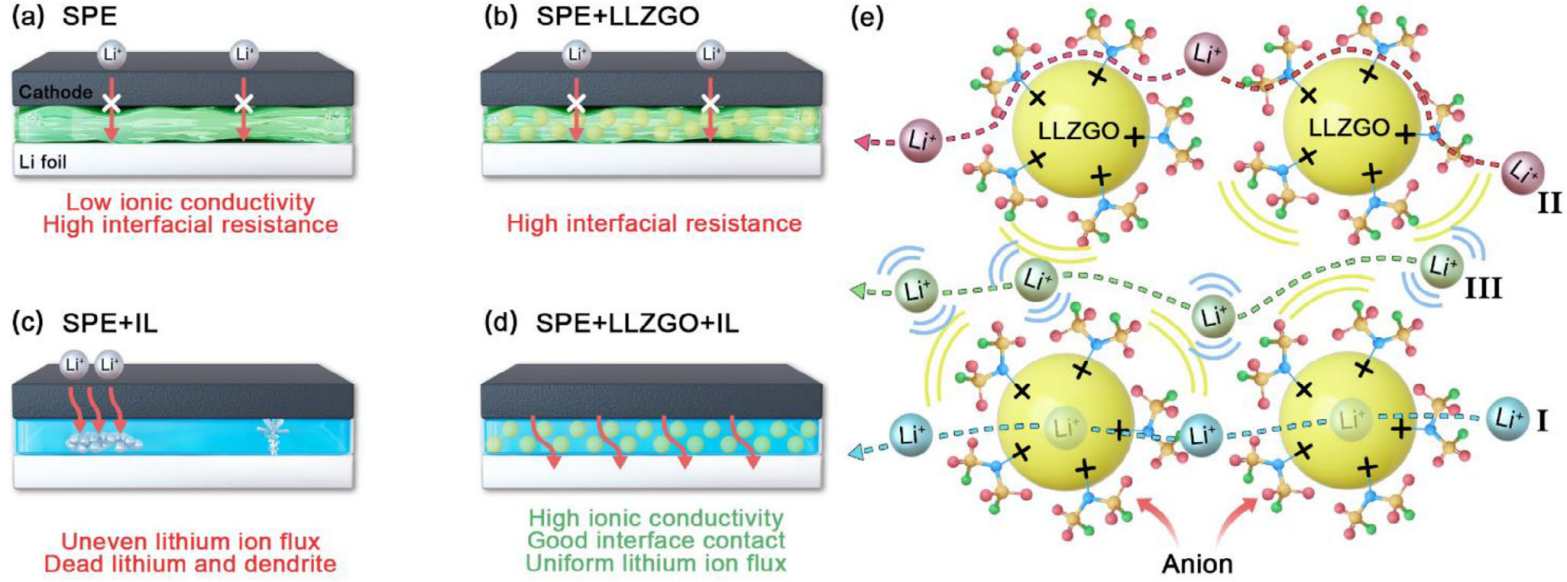
Schematics of working mechanisms for NCM‐811||Li cells with a) SPE, b) SPE+LLZGO, c) SPE+IL, and d) SPE+LLZGO+IL electrolytes. e) Li^+^ transport pathways within SPE+LLZGO+IL SSE.

**Figure** **6**a–d shows the mechanistic schemes of various SSEs. The SPE is limited by its inherently low ionic conductivity and high interfacial resistance. Although the incorporation of LLZGO into the SPE improves the ionic conductivity and *t*_Li_^+^, its effects are marginal. For SPE+IL, although the IL addition helps soften the SSE and alleviate interfacial discontinuity, the absence of LLZGO particles makes it challenging to maintain mechanical robustness and regulate the Li^+^ flux. This leads to uneven Li deposition and the formation of Li dendrites, which reduce cell durability. Of note, SPE+LLZGO+IL combines the benefits of LLZGO particles and the IL. High ionic conductivity and *t*_Li_^+^ and low interfacial resistance can be simultaneously achieved. Thus, homogenous Li deposition and a minimal amount of Li dendrite formation are found. Figure 6e visualizes the Li^+^ transport pathways within SPE+LLZGO+IL, showing the synergy between the LLZGO and the IL. Li^+^ ions can pass through the LLZGO particles (Pathway I), benefiting from the intrinsically good conductivity of LLZGO (i.e., ≈6.3 × 10^−4^ S cm^−1^).^[^ ^60^ ^]^ In addition, Li^+^ ions can migrate along the LLZGO surface space‐charge regions (Pathway II),^[^ ^61^ ^]^ which act as Li^+^ conduction highways. This study emphasizes another Li^+^ transport mechanism (Pathway III), where the positively charged LLZGO particles (due to acidic groups and/or oxygen vacancies) attract electrolyte anions, promoting Li^+^/anion dissociation and free Li^+^ migration within the SSE. These mechanisms are supported by the *t*_Li_^+^, EIS, and charge–discharge data above, highlighting the great potential of this thin SPE+LLZGO+IL SSE for high‐performance SSLBs and showcasing a model system with clear synergistic effects between LLZGO particles and an IL.

**Figure 7:**
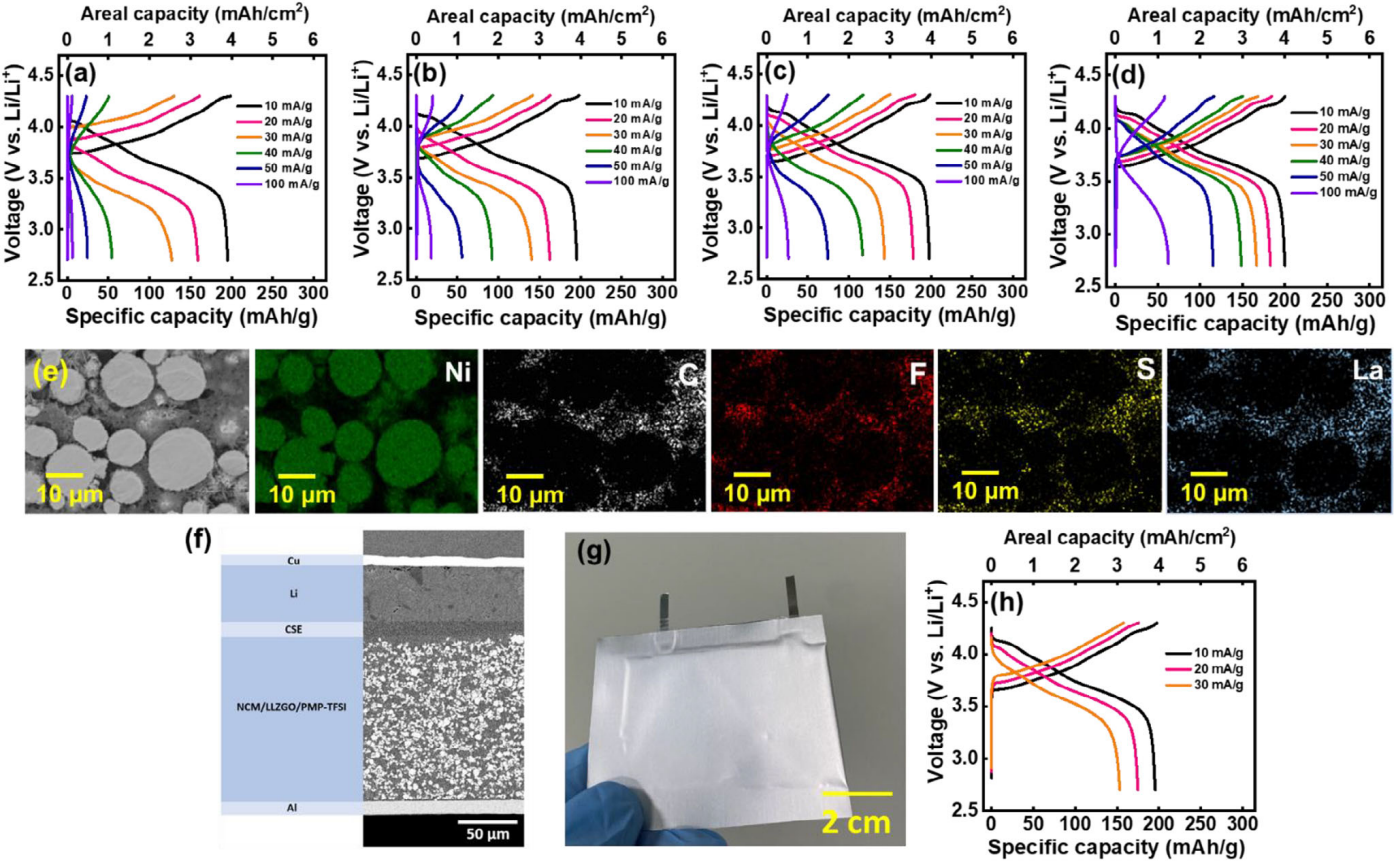
Galvanostatic charge–discharge curves of high‐mass‐loading (20 mg cm^−2^) a) NCM, b) NCM/LLZGO, c) NCM/PMP‐TFSI, and d) NCM/LLZGO/PMP‐TFSI cells. e) Top‐view EDS mapping images of NCM/LLZGO/PMP‐TFSI composite cathode. f) Cross‐section SEM image of NCM/LLZGO/PMP‐TFSI||SSE||Li full cell. g) Optical appearance and h) charge–discharge curves of NCM/LLZGO/PMP‐TFSI||SSE||Li pouch cell.

To achieve a high‐energy‐density SSLB, besides a thin, robust, and high‐conductivity SSE, it is crucial to develop a thick cathode with a high areal capacity. Herein, we try to develop a composite cathode (NCM‐811 plus ionic conductors) with an active material loading of ≈20 mg cm^−2^. The SPE+LLZGO+IL SSE was used. Three types of composite cathode were prepared, namely NCM/LLZGO, NCM/PMP‐TFSI, and NCM/LLZGO/PMP‐TFSI. The Li^+^ conductor content, including LLZGO and PMP‐TFSI, was fixed at 5 wt.%. The NCM/LLZGO/PMP‐TFSI cathode had 2.5 wt.% LLZGO and 2.5 wt.% PMP‐TFSI. PMP‐TFSI was selected due to its wide electrochemical stability window and the low corrosivity of the TFSI⁻ anion toward the Al cathode current collector.^[^ ^62^, ^63^ ^]^ A cathode without addition of a Li^+^ conductor (denoted as NCM) was also examined for comparison. The cells were tested at 30 °C at various current densities and the charge–discharge profiles were recorded. **Figure** **7**a shows the obtained data for the NCM cell. Because of the lack of effective Li^+^ conduction pathways within the cathode, the reversible capacity at high rate is limited. When LLZGO particles were incorporated into the high‐mass‐loading cathode to enhance Li^+^ transport, the cell rate capability improved, as shown in Figure 7b. The data in Figure 7c indicate that the introduction of PMP‐TFSI IL more effectively promotes the charge–discharge performance compared to adding LLZGO, which is likely related to the improved interfacial contact due to the softness of the PMP‐TFSI IL. Figure 7d shows the charge–discharge curves of the NCM/LLZGO/PMP‐TFSI cell, which outperformed all other cells. This configuration achieves an areal capacity of ≈4 mAh cm^−2^ at room temperature, which exceeds all reported values for oxide‐based composite cathodes for CSE‐based SSLBs, as shown in **Table** **3**. The measured capacities at various rates for the four types of cell are compared in **Table** **4**. The results suggest that the synergistic effects between LLZGO particles and the IL also appear in the high‐mass‐loading composite cathode (the mechanism is discussed later). Figure 7e shows the top‐view EDS mapping data of the NCM/LLZGO/PMP‐TFSI composite cathode, which confirm the formation of continuous Li^+^ conduction pathways made by LLZGO and the PMP‐TFSI IL. The cross‐section SEM image of the NCM/LLZGO/PMP‐TFSI||SSE||Li full cell is shown in Figure 7f. The thickness of the composite cathode, which is robust and compact, is as high as 125 µm. Excellent contact of the thin SSE with both the composite cathode and Li metal anode was verified. To assess the potential for practical applications, an NCM/LLZGO/PMP‐TFSI||SSE||Li pouch cell was assembled, as shown in Figure 7g. The charge–discharge curves of the pouch cell, shown in Figure 7h, reveal that the electrochemical performance closely resembles that of a coin cell. This suggests that the proposed SSE and composite cathode, which are effective in small‐scale coin cells, maintain their efficacy in larger, more practical pouch cells.

**Table 3:** Properties of oxide‐based composite cathodes for CSE‐based SSLBs reported in the literature.

Active material [AM]	AM loading [mg cm^−2^]	Current density	Oxide in composite cathode	Polymer and oxide in CSE	CSE thickness [µm]	Ionic conductivity of CSE [S cm^−1^]	Reversible capacity [mAh g^−1^]	Operation temperature [°C]	Areal capacity [mAh cm^−2^]	Refs.
LiNi_0.4_Co_0.2_Mn_0.4_O_2_	5	0.1C	Li_6.25_Al_0.25_La_3_Zr_2_O_12_	Polyethylene oxide (PEO) Li_6.25_Al_0.25_La_3_Zr_2_O_12_	≈100	7.59 × 10^−6^ at 25°C 1.93 × 10^−3^ at 70°C	122	70	0.61	[29]
LiNi_0.8_Co_0.1_Mn_0.1_O_2_	5	0.1C	La_2_(Ni_0.5_Li_0.5_)O_4_	PEO Li_7_La_3_Zr_2_O_12_	–	–	144	70	0.72	[30]
LiNi_0.6_Co_0.2_Mn_0.2_O_2_	10.6	0.05C	Li_7_La_3_Zr_2_O_12_	PEO Li_7_La_3_Zr_2_O_12_	≈40	7.6 × 10^−5^ at 25°C 3.0 × 10^−4^ at 70°C	150	70	1.60	[31]
LiNi_0.8_Co_0.15_Al_0.05_O_2_	2–3	9 mA g^−1^	Li_1.3_Al_0.3_Ti_1.7_(PO_4_)_3_	PEO Li_1.3_Al_0.3_Ti_1.7_(PO_4_)_3_ Li_7_La_3_Zr_2_O_12_	80–100	1.0 × 10^−4^ at 25°C	184	60	0.37–0.55	[32]
LiFePO_4_	7	0.1C	Li_7_La_3_Zr_2_O_12_	PEO Li_7_La_3_Zr_2_O_12_	≈40	5.1 × 10^−6^ at 30°C 1.7 × 10^−4^ at 70°C	144	70	1.00	[33]
LiFePO_4_	3	0.2C	Li_1.3_Al_0.3_Ti_1.7_(PO_4_)_3_	PEO Li_1.3_Al_0.3_Ti_1.7_(PO_4_)_3_	≈80	9.5 × 10^−6^ at 30°C 1.6 × 10^−3^ at 80°C	130	80	0.39	[34]
LiNi_0.6_Co_0.2_Mn_0.2_O_2_	7	0.05C	Li_6.25_Ga_0.25_La_3_Zr_2_O_12_	Polyacrylonitrile Li_6.25_Ga_0.25_La_3_Zr_2_O_12_	≈80	4.59 × 10^−4^ at 25°C	143	25	1.00	[35]
LiNi_0.6_Co_0.2_Mn_0.2_O_2_	2	0.02C	Li_7_La_3_Zr_2_O_12_	PEO Li_7_La_3_Zr_2_O_12_	≈100	4.42 × 10^−4^ at 55°C	166	55	0.33	[36]
LiNi_0.8_Co_0.1_Mn_0.1_O_2_	20	10 mA g^−1^ (0.05C)	Li_6.25_Ga_0.25_La_3_Zr_2_O_12_	PVDF‐HFP PPC Li_6.25_Ga_0.25_La_3_Zr_2_O_12_	≈18	8.6 × 10^−4^ at 30°C	200	30	4.00	This study

**Table 4:** Reversible capacities of NCM‐811 measured at various rates for various high‐mass‐loading (20 mg cm^−2^) composite cathodes.

Current density [mA g^−1^]	Reversible capacity [mAh g^−1^]			
	NCM	NCM/LLZGO	NCM/PMP‐TFSI	NCM/LLZGO/PMP‐TFSI
10	195	196	198	200
20	158	162	178	183
30	127	140	143	166
40	52	92	116	148
50	23	55	74	115
100	5	18	25	60

The effects of LLZGO and PMP‐TFSI content in the cathode layer on the electrochemical performance were examined. The obtained data are shown in Figure S10 (Supporting Information). When the content was either decreased (to 3 wt.% of LLZGO/PMP‐TFSI) or increased (to 7 wt.% of LLZGO/PMP‐TFSI), the charge–discharge capacities of NCM‐811 deteriorated. An excessive amount of LLZGO/PMP‐TFSI can hinder the electron conduction within the cathode,^[^ ^64^ ^]^ leading to the performance decay. Therefore, 5 wt.% LLZGO/PMP‐TFSI was used for further investigation.

**Figure 8:**
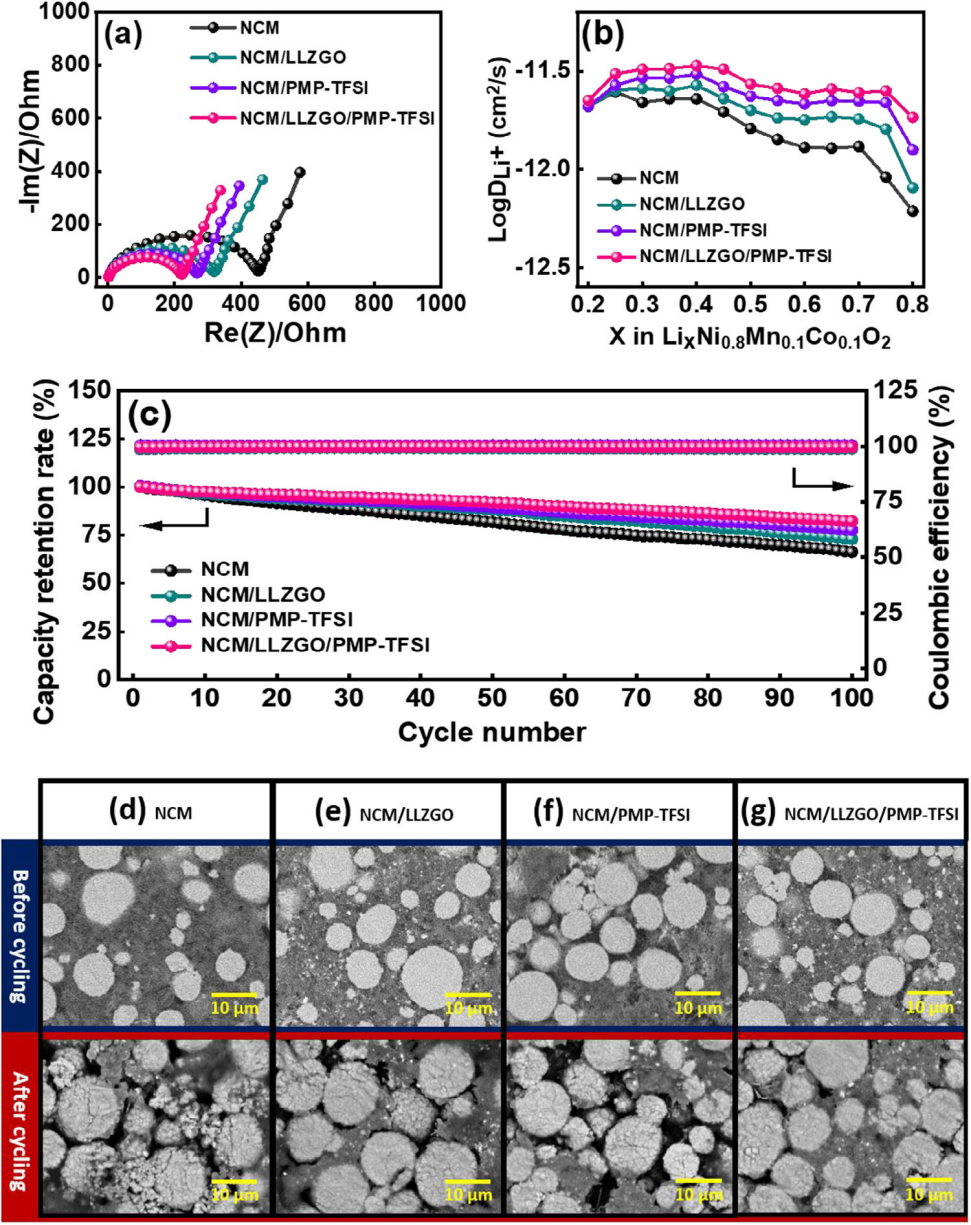
a) EIS spectra of various cathodes. b) *D*_Li_^+^ values of various composite cathodes measured using GITT. c) Cycling stability of full cells with various cathodes measured at 20 mA g^−1^. Graphite anodes are used to achieve a negative‐to‐positive electrode capacity ratio of 1.1. The cross‐section SEM images of d) NCM, e) NCM/LLZGO, f) NCM/PMP‐TFSI, and g) NCM/LLZGO/PMP‐TFSI cathodes before and after cycling.

To further study the electrochemical characteristics of the four types of high‐mass‐loading cathode, EIS and galvanostatic intermittent titration technique (GITT) measurements were performed. The EIS results in **Figure** **8**a reveal that the charge transfer resistance (*R*_ct_) values for the NCM, NCM/LLZGO, NCM/PMP‐TFSI, and NCM/LLZGO/PMP‐TFSI electrodes are 446, 315, 263, and 219 Ω, respectively. The NCM/LLZGO/PMP‐TFSI cell having the lowest *R*_ct_ value is attributed to the formation of effective Li^+^ conduction channels, which intimately connect the NCM‐811 electroactive material and facilitate redox reactions. The rigid LLZGO ceramic particles alone cannot well form a continuous Li^+^ pathway, whereas the PMP‐TFSI IL alone cannot maintain the mechanical strength of the electrode. The combination of LLZGO and PMP‐TFSI forms an ionogel,^[^ ^65^ ^]^ which is distributed within the cathode and facilitates charge–discharge processes. The apparent Li^+^ diffusion coefficient (*D*_Li_^+^) within the cathode can be calculated based on the GITT data (see Supporting Information).^[^ ^66^ ^]^ Figure 8b shows the *D*_Li_^+^ values at various degrees of discharge derived from the voltage profiles presented in Figure S11 (Supporting Information). The obtained *D*_Li_^+^ values for the four types of cathode range from 6 × 10^−13^ to 4 × 10^−12^ cm^2^ s^−1^, which is consistent with the literature.^[^ ^67^, ^68^ ^]^ As the electroactive material undergoes phase changes at various states of discharge, the pathways for Li^+^ diffusion can be altered, leading to *D*_Li_^+^ variation. The average *D*_Li_^+^ values for the NCM, NCM/LLZGO, NCM/PMP‐TFSI, and NCM/LLZGO/PMP‐TFSI cathodes are 1.67 × 10^−12^, 2.03 × 10^−12^, 2.38 × 10^−12^, and 2.73 × 10^−12^ cm^2^ s^−1^, respectively. The low *R*_ct_ and high *D*_Li_^+^ values explain the superior rate capability of the NCM/LLZGO/PMP‐TFSI cell. A tape peel test was conducted to evaluate the adhesion strength of the four types of cathode. As shown in Figure S12 (Supporting Information), the NCM/PMP‐TFSI cathode exhibits noticeably poorer adhesion compared to the other electrodes. This mechanical drawback limits the Li^+^ conducting properties and cycling stability of the NCM/PMP‐TFSI cathode.

The cycling stability of various cathodes was also evaluated. Because of the high areal capacity (≈4 mAh cm^−2^), the Li metal anode cannot maintain durability due to large interface movement.^[^ ^69^ ^]^ Therefore, graphite anodes were paired with the various types of cathode, with a negative‐to‐positive electrode capacity ratio of 1.1. Figure 8c shows the results measured at a rate of 20 mA g^−1^. The data show that the NCM, NCM/LLZGO, NCM/PMP‐TFSI, and NCM/LLZGO/PMP‐TFSI cells retained 66%, 72%, 75%, and 82%, respectively, of their initial capacities after 100 charge–discharge cycles. The root causes of the capacity deterioration were examined using cross‐section SEM. Figure 8d,e shows the morphology change of the NCM and NCM/LLZGO electrodes, respectively. Significant cracking and pulverization of the NCM‐811 particles were observed after cycling. This degradation can be attributed to the lack of smooth Li^+^ conduction channels, which causes non‐uniform lithiation/delithiation and thus inhomogeneous volume variation of the active material particles. Moreover, there is no soft buffer layer (such as PMP‐TFSI) within the structures. Thus, the stress/strain generated during cycling is hardly released, leading to mechanical collapse. In contrast, the incorporation of PMP‐TFSI into the NCM/PMP‐TFSI cathode helps mitigate volume changes of the electroactive material; however, it degrades the mechanical integrity of the electrode, especially after cycling (Figure 8f). The structural flaws can lead to discontinuity of both electronic and ionic conduction, deteriorating electrode performance. As shown in Figure 8g, the NCM/LLZGO/PMP‐TFSI cathode has the optimal structural stability, which contributes to the best capacity retention after charge–discharge cycling, as found in Figure 8c. The LLZGO/PMP‐TFSI ionogel functions as both a Li^+^‐conducting agent and an effective cushion material within the thick composite cathode. As a result, the NCM/LLZGO/PMP‐TFSI electrode achieved superior rate capability and cycling stability.

**Figure 9:**
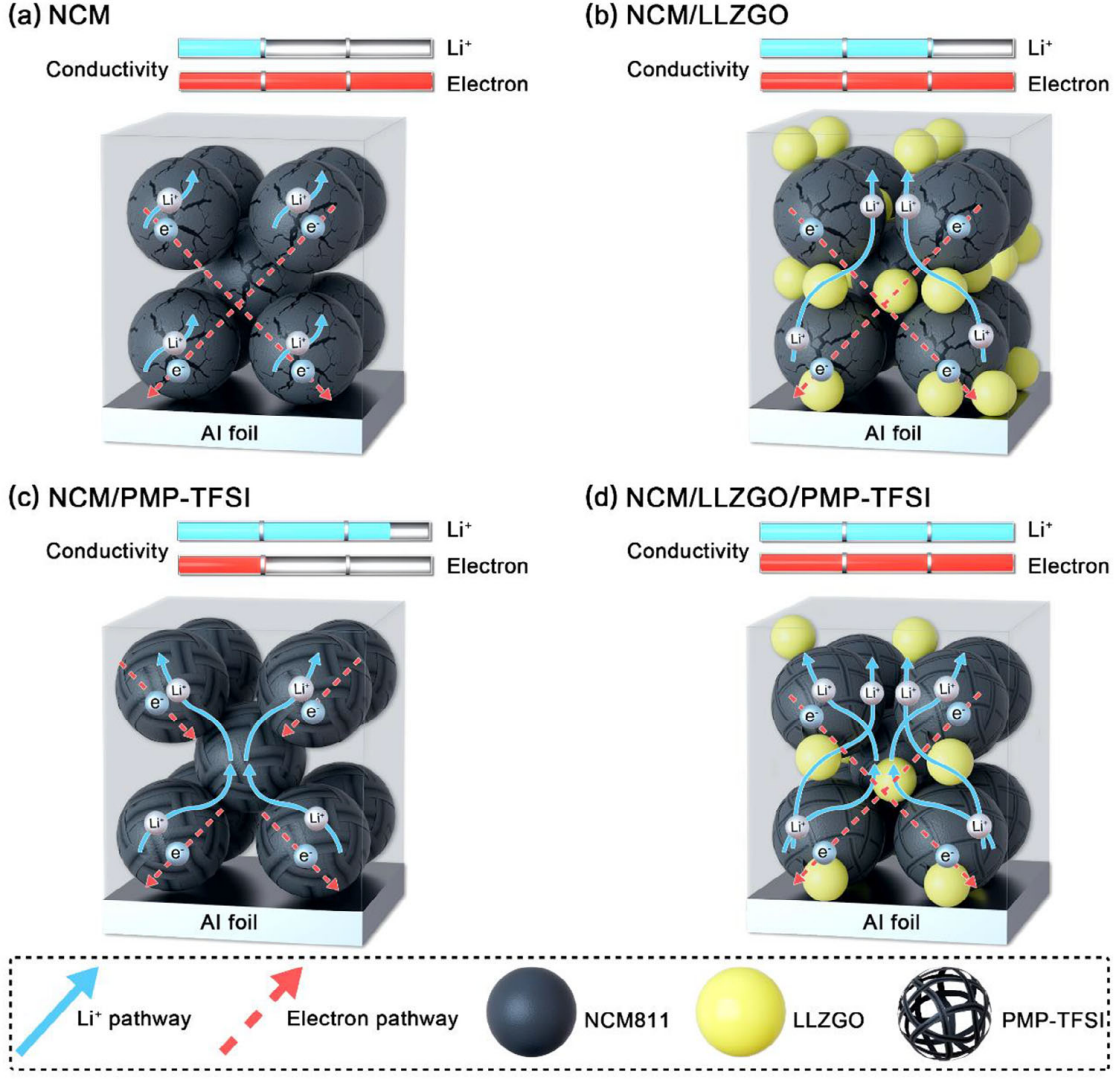
Schematics of working mechanisms for a) NCM, b) NCM/LLZGO, c) NCM/PMP‐TFSI, and d) NCM/LLZGO/PMP‐TFSI cathodes.

**Figure** **9** shows the working mechanisms of the NCM, NCM/LLZGO, NCM/PMP‐TFSI, and NCM/LLZGO/PMP‐TFSI cathodes. The NCM electrode without the incorporation of extra Li^+^ conductors shows limited electroactivity due to insufficient Li^+^ transport. Although the NCM/LLZGO electrode has slightly higher Li^+^ conductivity, the improvement is marginal because the stiff and disperse LLZGO particles hardly form continuous conduction pathways. On the other hand, a superior Li^+^ conduction framework is established within the NCM/PMP‐TFSI; however, the IL phase wrapping the NCM‐811 particles can cause structural instability and block electron transport pathways, restricting the electrode's high‐rate performance. Of note, the NCM/LLZGO/PMP‐TFSI composite cathode benefits from the synergistic effects between the two kinds of Li^+^ conductor and thus shows the optimal electrochemical properties. The LLZGO/PMP‐TFSI ionogel can adequately glue the NCM‐811 and conducting carbon, securing both the ionic and electronic conduction of the electrode. Moreover, this soft and adhesive phase can accommodate the active material volume variation during cycling and stabilize the cathode/SSE interface, both of which are favorable for cell durability.

We believe that the proposed approach for constructing the composite cathode can be extended to other cathode materials. To demonstrate this, we fabricated a LiCoO_2_/LLZGO/PMP‐TFSI cathode. As shown in Figure S13 (Supporting Information), this composite electrode exhibits superior charge–discharge properties compared to a plain LiCoO_2_ cathode in an SPE+LLZGO+IL SSE cell. Although further optimization of the electrode composition may be necessary for different types of cathodes, the concept of incorporating this ISE/IL‐combined Li⁺ conductor is broadly applicable to SSLBs.

**Figure 10:**
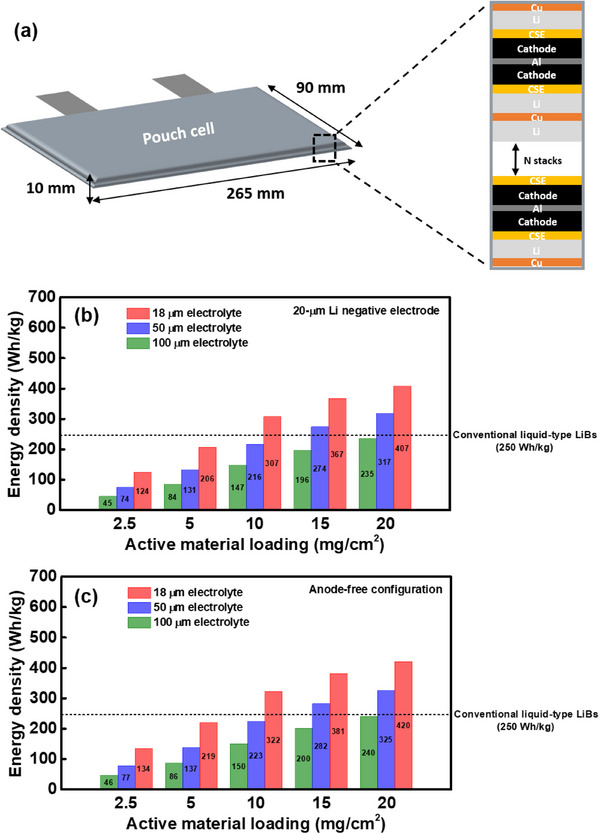
a) Specifications and configurations of the multi‐layer pouch cell used to estimate SSLB energy density. The calculated energy densities of pouch cells with various active material loading amounts and CSE thicknesses, while using b) 20‐µm Li negative electrode and c) anode‐free configuration.

To further justify the importance of the developed thin PE‐scaffold SSE and thick composite cathode for SSLBs, we estimate the energy densities of NCM/LLZGO/PMP‐TFSI||SSE||Li pouch cells with various parameter values. The multi‐layer pouch cell is illustrated in **Figure** **10**a. It is 265 mm in length, 90 mm in width, and 10 mm in thickness, matching the dimensions of a commercial cell made by SK Innovation.^[^ ^70^ ^]^ Figure 10b shows the calculation results based on a 20‐µm Li negative electrode and other cell parameters (such as those of the separator, current collectors, packing materials, and tabs) given in the literature.^[^ ^70^ ^]^ As shown, if a 100‐µm SSE and 2.5 mg cm^−2^ cathode loading, which are commonly used in the literature, are adopted, the SSLB has an energy density of only ≈45 Wh kg^−1^. This value is much lower than that (≈250 Wh kg^−1^) of a commercial state‐of‐the‐art LiB cell with a conventional liquid electrolyte.^[^ ^71^ ^]^ Thus, the thick SSE and thin cathode are rather impractical. As shown, reducing the SSE thickness clearly increases the cell energy density, since the portion of the electro‐inactive SSE is reduced and thus more active materials can be packed within the cell. The calculation results suggest that with an 18‐µm SSE, if the NCM‐811 loading is 10 mg cm^−2^, the cell energy density (≈307 Wh kg^−1^) can surpass that of the above‐mentioned liquid‐electrolyte cell. Of note, the proposed 18‐µm PE‐scaffold SSE membrane and 20 mg cm^−2^ cathode can lead to a projected energy density of ≈407 Wh kg^−1^. The ultimate goal in developing high‐energy‐density SSLBs is to use an anode‐free design (no metallic Li is used in a fresh cell). Therefore, we also calculated the energy densities of anode‐free cells with various SSE thicknesses and cathode loading amounts; the obtained results are shown in Figure 10c. The projected energy density reaches ≈420 Wh kg^−1^ for the SSE and composite cathode developed in this study, demonstrating the significant potential of these components for high‐performance SSLB applications.

## 3 Conclusion

This study developed an 18‐µm scaffold‐supported CSE that incorporates an IL. The flexible and robust CSE exhibits a competitive Li^+^ conductivity of 8.6 × 10^−4^ S cm^−1^ at 30 °C. The synergistic effects between LLZGO particles and an IL in a polymer matrix were demonstrated. These effects decreased the *R*_e_ and *R*_int_ of the CSE and led to superior electrochemical performance of the constructed NCM‐811||CSE||Li cell. To increase the cell energy density, a high‐areal‐capacity cathode was developed. The simultaneous incorporation of LLZGO and PMP‐TFSI IL, which form an ionogel, into a thick composite cathode is a novel approach. The combination of these ionic conductors not only establishes continuous and effective Li^+^ conduction pathways but also buffers the active material volume variation during charging and discharging. With an NCM‐811 mass loading of ≈20 mg cm^−2^ and an operation temperature of 30 °C, the composite cathode shows a high specific capacity of 200 mAh g^−1^ and a great areal capacity of ≈4 mAh cm^−2^. When the composite cathode was paired with a graphite anode to form a solid‐state full cell, 82% capacity retention was achieved after 100 cycles. The anode‐free NCM/LLZGO/PMP‐TFSI||CSE pouch cell is projected to have an impressive energy density of 420 Wh kg^−1^, which is considerably higher than that of a state‐of‐the‐art liquid‐electrolyte LiB. The integration of an IL and LLZGO into both the CSE and the high‐mass‐loading cathode, along with the scalable fabrication methods, highlight the practical viability of this work. The results demonstrate the unique functions of an IL in boosting the performance of SSEs and composite cathodes and provide a scalable and feasible cell configuration for realizing cost‐effective and high‐energy‐density SSLBs.

## 4 Experimental Section

### Preparation of LLZGO Powder

LLZGO powder was synthesized using a solid‐state‐reaction method. To remove moisture, the Li_2_CO_3_ (Acros Organics, 99%), La_2_O_3_ (Acros Organics, 99.99%), ZrO_2_ (Strem Chemicals, 99%), and Ga_2_O_3_ (Alfa Aesar, 99.99%) raw materials were preheated at 100 °C for 12 h. The obtained Li_2_CO_3_, La_2_O_3_, ZrO_2_, and Ga_2_O_3_ were weighed according to the stoichiometry of Li_6.25_La_3_Zr_2_Ga_0.25_O_12_. An additional 10 wt.% Li_2_CO_3_ was added to compensate for the lithium loss during calcination. The particles were ball‐milled for 4 h at 300 rpm to achieve homogeneity, followed by heating at 900 °C for 12 h under air. After cooling down in the furnace, LLZGO powder was obtained.

### Preparation of SSE

The IL was prepared by dissolving 1 M LiTFSI (TCI, 98%) in PMP‐FSI (Solvionic, 99.9%). In addition, 1 wt.% LiDFOB (Sigma–Aldrich, 99%) additive was used. PVDF‐HFP (Mw: 400 000 g mol^−1^) and PPC (Mw: 50 000 g mol^−1^) were purchased from Sigma–Aldrich. Dimethylformamide (DMF, 99.8%) was purchased from J.T. Baker. Four kinds of SSE were prepared in this study. The SPE slurry was prepared by mixing PVDF‐HFP, PPC, and LiTFSI in a weight ratio of 15:1.7:83.3 in DMF. The SPE+LLZGO slurry was prepared by mixing SPE slurry with 10 wt.% LLZGO. For the SPE+IL slurry, the SPE slurry was mixed with the IL in a mass ratio of 2:1. The SPE+LLZGO+IL slurry was prepared by mixing the SPE+LLZGO slurry with the IL in a mass ratio of 2:1. PE (W‐SCOPE Corporation) membranes (thickness = 16 µm; porosity = 43%) were used as the scaffolds, which were then infiltrated with various electrolyte slurries. Finally, the SSE membranes were dried in a vacuum oven at 70 °C for 24 h to completely remove the DMF solvent. A custom‐built tabletop tensile testing machine was employed to evaluate the mechanical properties of the SSE membranes with a tensile rate of 0.05 mm per second.

### Preparation of NCM‐811 Composite Cathodes

The NCM cathode slurry was prepared by mixing 87 wt.% NCM‐811 (UBIQ Technology Co., Ltd.), 5 wt.% Super P, 5 wt.% vapor‐grown carbon fibers (VGCF, Industrial Technology Research Institute, Taiwan), and 3 wt.% poly(vinylidene fluoride) (PVDF) binder in *N*‐methyl‐*2*‐pyrrolidinone (NMP, Alfa Aesar, 99%). To prepare the NCM/LLZGO composite cathode slurry, 82 wt.% NCM‐811, 5 wt.% SP, 5 wt.% VGCF, 5 wt.% LLZGO, and 3 wt.% PVDF were mixed in NMP. For the NCM/PMP‐TFSI composite cathode slurry, 82 wt.% NCM‐811, 5 wt.% SP, 5 wt.% VGCF, 5 wt.% PMP‐TFSI (Solvionic, 99.9%), and 3 wt.% PVDF were mixed in NMP. The NCM/LLZGO/PMP‐TFSI slurry consisted of 82 wt.% NCM‐811, 5 wt.% SP, 5 wt.% VGCF, 2.5 wt.% LLZGO, 2.5 wt.% PMP‐TFSI, and 3 wt.% PVDF in NMP. All slurries were stirred thoroughly and cast onto aluminum foil using a doctor blade. After casting, the cathodes were dried in a vacuum oven at 70 °C for 12 h. For the graphite anodes, the electrode slurry was prepared by mixing 80 wt.% graphite (China Steel Chemical Corporation), 5 wt.% SP, 5 wt.% VGCF, and 10 wt.% PVDF in NMP. This slurry was coated onto Cu foil and vacuum‐dried at 90 °C for 12 h.

### Material and Electrochemical Characterizations

The crystallinity of the LLZGO powder was characterized by XRD (Bruker D2 Phaser). Cu Kα radiation was used as the X‐ray source. The TEM analyses were performed with a Chemi‐STEM microscope (FEI Titan). The morphology and chemical composition of the samples were examined using SEM (JEOL JSM‐6700F) and the auxiliary EDS. The cross‐section samples were prepared using an SEM Mill (Fischione Instruments Model 1061) or an FIB (FEI VERSA 3D). To probe the thermal properties of various SSEs, DSC (NETZSCH 3500 Sirius) measurements were conducted under an N_2_ atmosphere. The samples were scanned from 50 to 200 °C at a heating rate of 10 °C min^−1^. Zeta potential measurements were conducted with a Zeta potential analyzer (Otsuka Electronics ELSZ‐2000). For the evaluation of electrochemical properties, the electrodes were punched to match the required dimensions of a CR2032 coin cell. Li metal foil was used as the counter electrode. The coin cells were assembled in an Ar‐filled glove box (O_2_: <0.1 ppm, H_2_O: <0.1 ppm). The pouch cells were fabricated in a dry room with a dew point of ≈–50 °C. The EIS measurements were conducted using a BioLogic VSP‐300 workstation in a frequency range of 7 × 10^6^–1 × 10^−1^ Hz with a voltage perturbation amplitude of 10 mV. The electrochemical performance (such as charge–discharge capacity, rate capability, and cycling stability) and GITT measurements were performed at 30 °C using NEWARE CT‐4000 battery testers.

### Statistical Analysis

The EIS, GITT, and charge–discharge measurements of various electrodes were repeated at least five times to ensure validity. The data deviation was typically within ≈3%. The reported values were the medians. For XRD data, background subtraction and phase identification were conducted using the EVA and TOPAS programs provided in the Bruker software package. Origin software was used for data analysis and processing.

## Conflict of Interest

The authors declare no conflict of interest.

## Supporting information



Supporting Information

## Data Availability

The data that support the findings of this study are available from the corresponding author upon reasonable request.
