# Supplementary material for: Ionic Liquid Enabled High‐Energy‐Density Solid‐State Lithium Batteries with High‐Areal‐Capacity Cathode and Scaffold‐Supported Composite Electrolyte
Source: Small. 2025 Jul 26;21(37):e03865. doi: 10.1002/smll.202503865 (PMC12444873; doi:10.1002/smll.202503865)
Supplement: Supplementary file 1 — Supporting Information [file SMLL-21-e03865-s001.docx]

Supporting Information

**Ionic Liquid Enabled High-Energy-Density Solid-State Lithium Batteries with High-Areal-Capacity Cathode and Scaffold-Supported Composite Electrolyte**

*Tzu-Yu Kuo, Jagabandhu Patra, Cheng-Chia Chen, Chun-Chen Yang, Chien-Nan Hsiao, Tsai-Fu Chung, Chung-Jen Tseng, Rajendra S. Dhaka, Chien-Te Hsieh, Ju Li, Jeng-Kuei Chang****

**Measurements of the lithium-ion transference number (*t*_Li_^+^)**

The Li^+^ transference number (*t*_Li_^+^) was determined by a dc polarization method combined with impedance spectroscopy, as reported by Bruce-Vincent,^[1,2]^ and was calculated according to Equation (i).

$t_{{Li}^{+}}= \frac{I_{ss}}{I_{0}}\left( \frac{\Delta V-I_{0}R_{0}}{\Delta V-I_{ss}R_{ss}} \right)$ (i)

Where, *I_o_* and *I_ss_* represent the initial current and steady-state polarization current, respectively. *ΔV* is the applied polarization voltage (10 mV). *R_o_* and *R_ss_* indicate the charge transfer resistance of the cell before and after polarization.

Reference

[1] J. Evans, C. A. Vincent, P. G. Bruce, *Polymer* 1987, *28*, 2324−2328.

[2] P. G. Bruce, J. Evans, C. A. Vincent, *Solid State Ionics* 1988, *28*−*30*, 918−922.

**Measurements of the apparent Li^+^ diffusion coefficient (*D*_Li_^+^)**

The apparent Li^+^ diffusion coefficient (*D*_Li_^+^) of the electrodes can be calculated based on GITT data.^[3]^ The following equation is used to evaluate the *D*_Li_^+^ values.

$D_{{Li}^{+}}= \frac{4}{\pi\tau}\left( \frac{n_{M}V_{M}}{S} \right)^{2}\left( \frac{{\Delta V}_{s}}{{\Delta V}_{t}} \right)^{2}$ (ii)

Where, *n_M_* and *V_M_* are the molar number (mol) and molar volume (cm^3^ mol^–1^) of the active material, respectively. S is the geometric surface area of the electrode, and τ is the time duration of the pulse. For the NCM-811, assuming the spherical particles having a radius of *R*_s_, Equation (ii) becomes:

$D_{{Li}^{+}}= \frac{4}{\pi\tau}\left( \frac{R_{s}}{3} \right)^{2}\left( \frac{{\Delta V}_{s}}{{\Delta V}_{t}} \right)^{2}$ (iii)

Reference

[3] Z. Shen, L. Cao, C. D. Rahn, C.-Y. Wang, *J. Electrochem. Soc.* 2013, *160*, A1842.

**
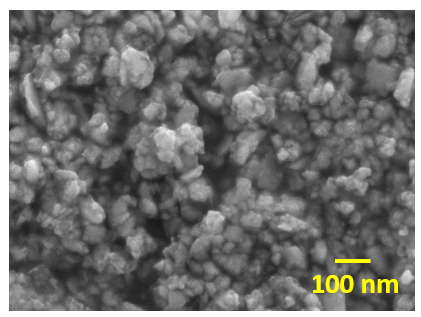
**

**Figure S1.** SEM image of LLZGO particles.

**
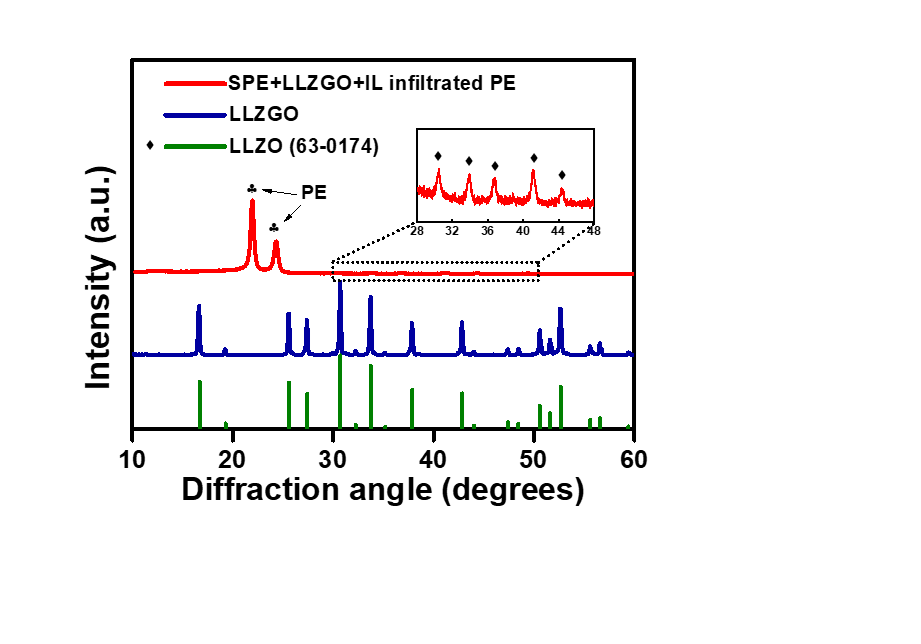
**

**Figure S2.** XRD patterns of LLZGO powder and SPE+LLZGO+IL infiltrated PE layer.

**
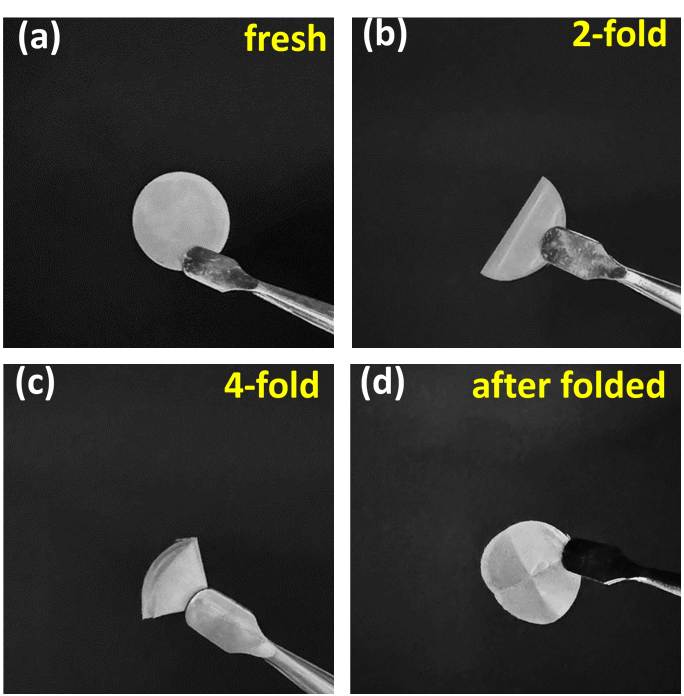
**

**Figure S3.** Optical appearance of (a) as-prepared, (b) 2-folded, (c) 4-folded, and (d) unfolded CSE layers.


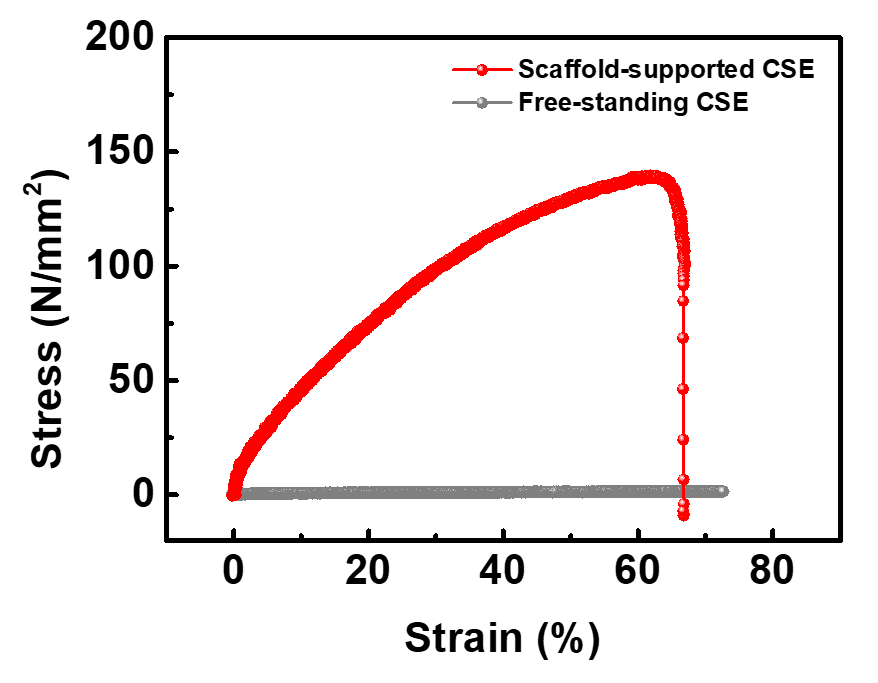


**Figure S4.** Tensile testing results of scaffold-supported CSE and free-standing CSE.

**Figure S5.** Arrhenius plots of ionic conductivity for various SSEs.

**Figure S6.** DSC data of SPE+IL and SPE+LLZGO+IL solid-electrolyte samples.


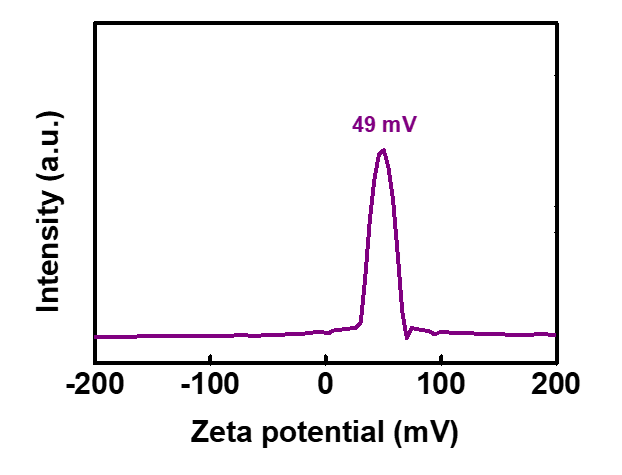


**Figure S7.** Zeta potential of LLZGO particles in PMP-FSI IL.


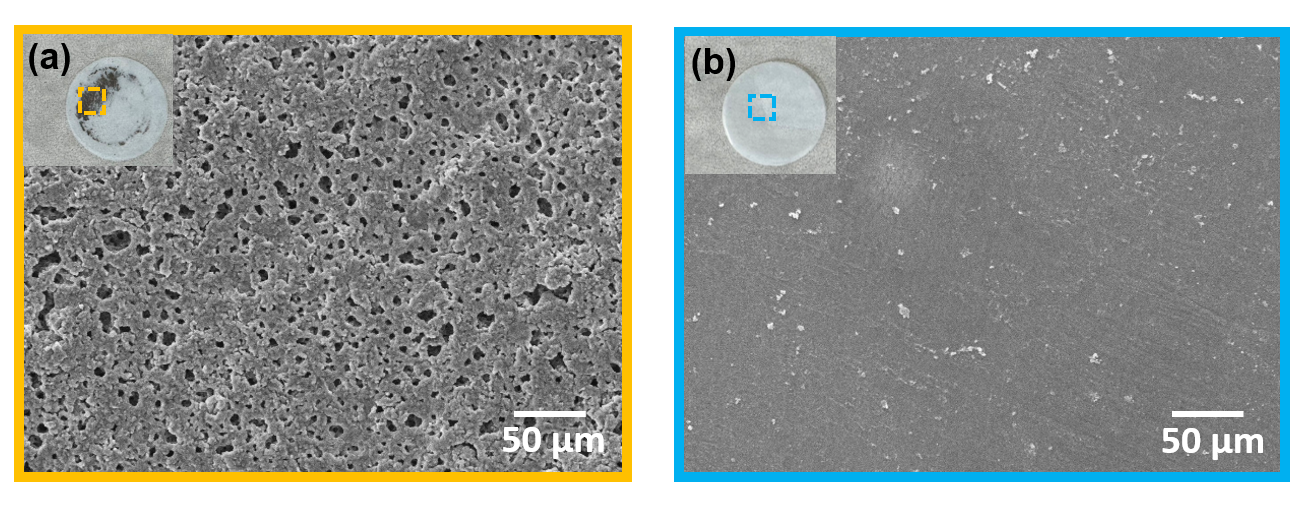


**Figure S8.** Optical and SEM images of (a) SPE+IL and (b) SPE+LLZGO+IL layers after 200 Li plating/stripping cycles.


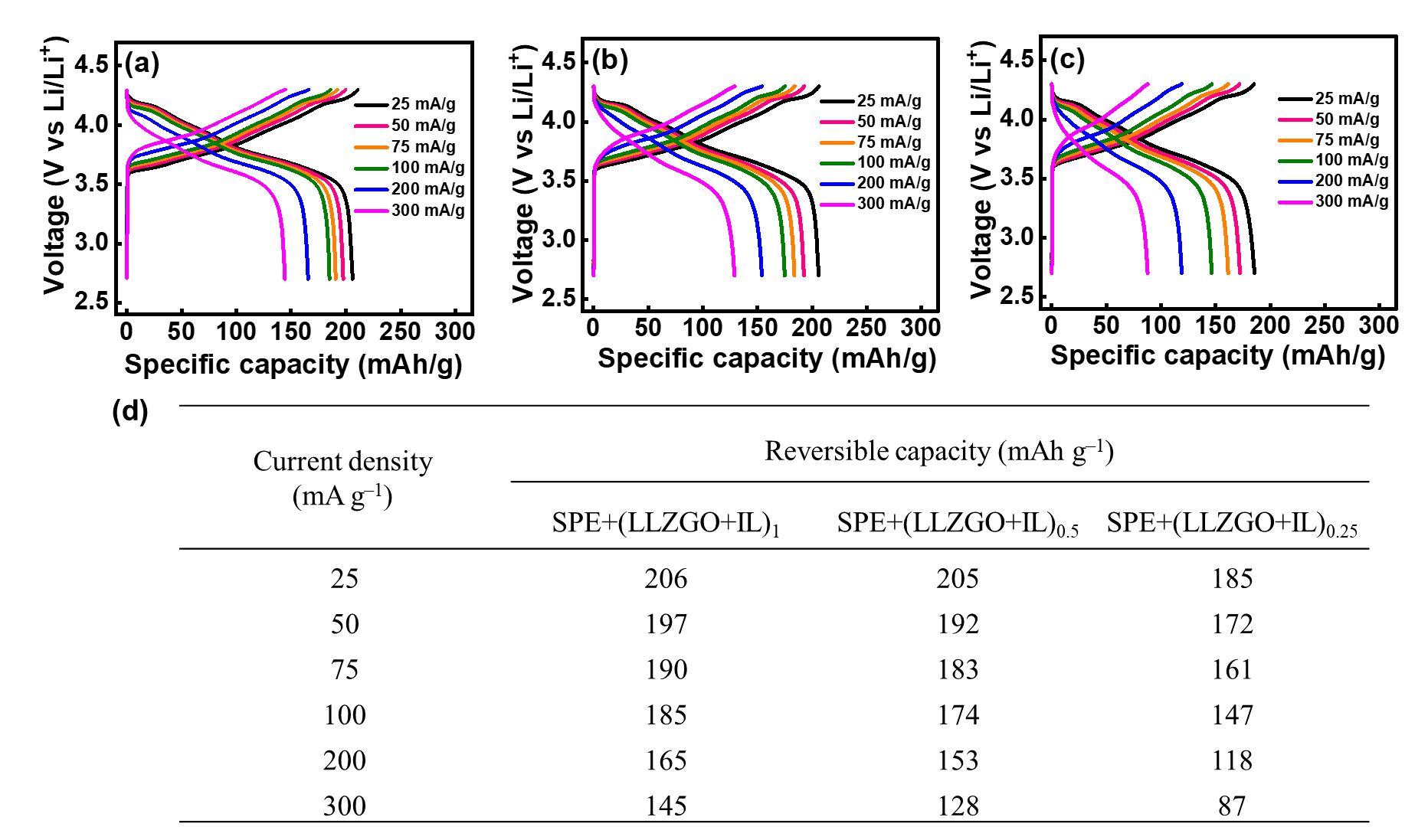


**Figure S9.** Galvanostatic charge-discharge curves of NCM-811||Li cells with (a) SPE+(LLZGO+IL)_1_, (b) SPE+(LLZGO+IL)_0.5_ and (c) SPE+(LLZGO+IL)_0.25_ electrolytes. (d) Comparison of reversible capacities of NCM-811 measured at different rates in various cells. SPE+(LLZGO+IL)_0.5_ indicates that the amounts of LLZGO and IL are reduced by half, while SPE+(LLZGO+IL)_0.25_ refers to a reduction to one-quarter of their original amounts.

**
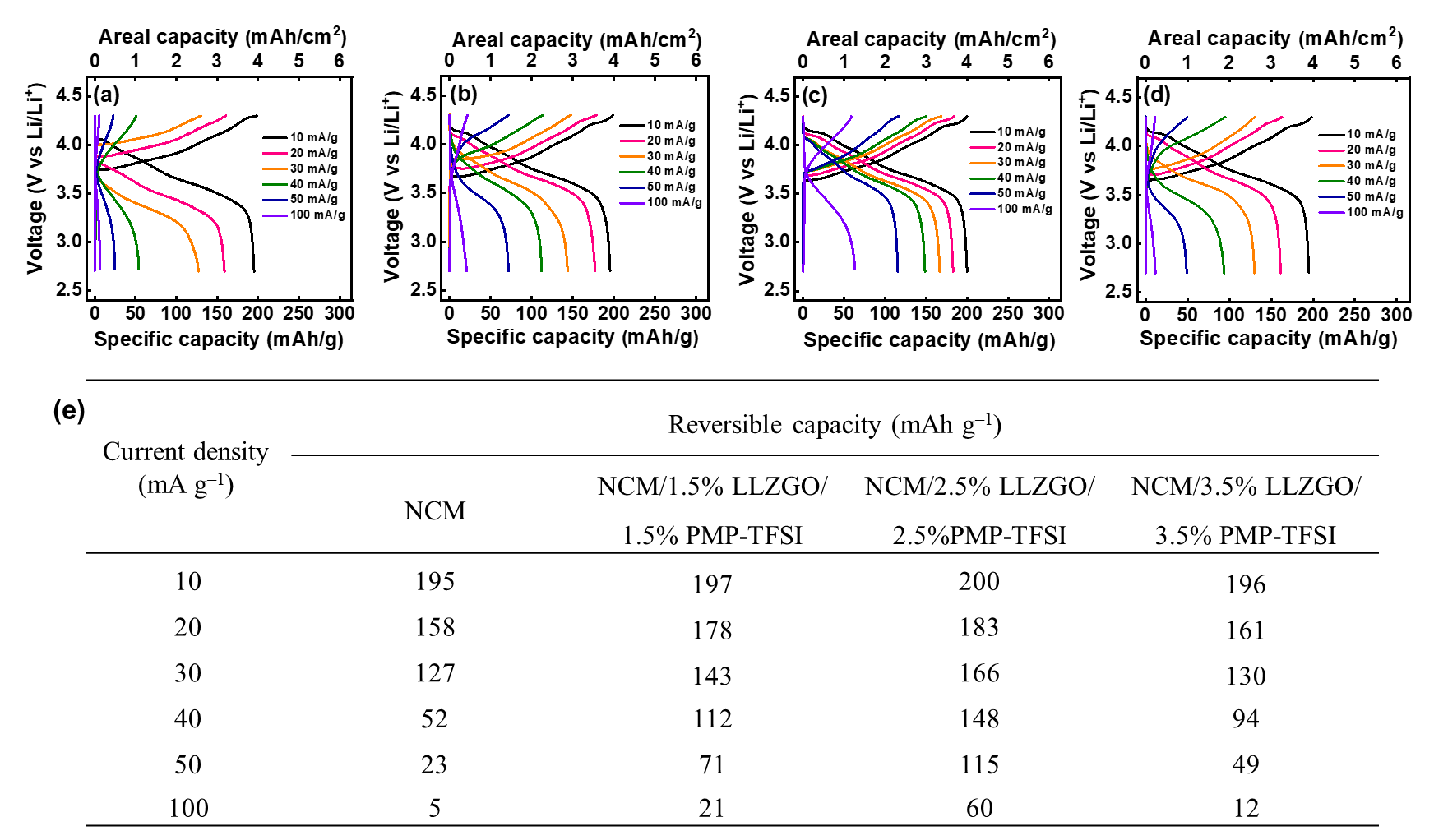
**

**Figure S10.** Galvanostatic charge-discharge curves of high-mass-loading (20 mg cm^–2^) (a) NCM, (b) NCM/1.5% LLZGO/1.5%PMP-TFSI, (c) NCM/2.5% LLZGO/2.5%PMP-TFSI, and (d) NCM/3.5% LLZGO/3.5%PMP-TFSI cells. (e) Comparison of reversible capacities at NCM-811 measured at different rates in various cells.

**Figure S11.** GITT data for four types of high-mass-loading cathode.


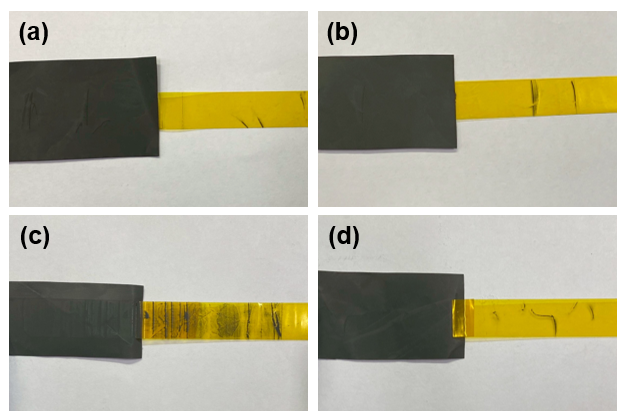


**Figure S12.** Tape adhesion test results of (a) NCM, (b) NCM/LLZGO, (c) NCM/PMP-TFSI, and (d) NCM/LLZGO/PMP-TFSI cathodes.


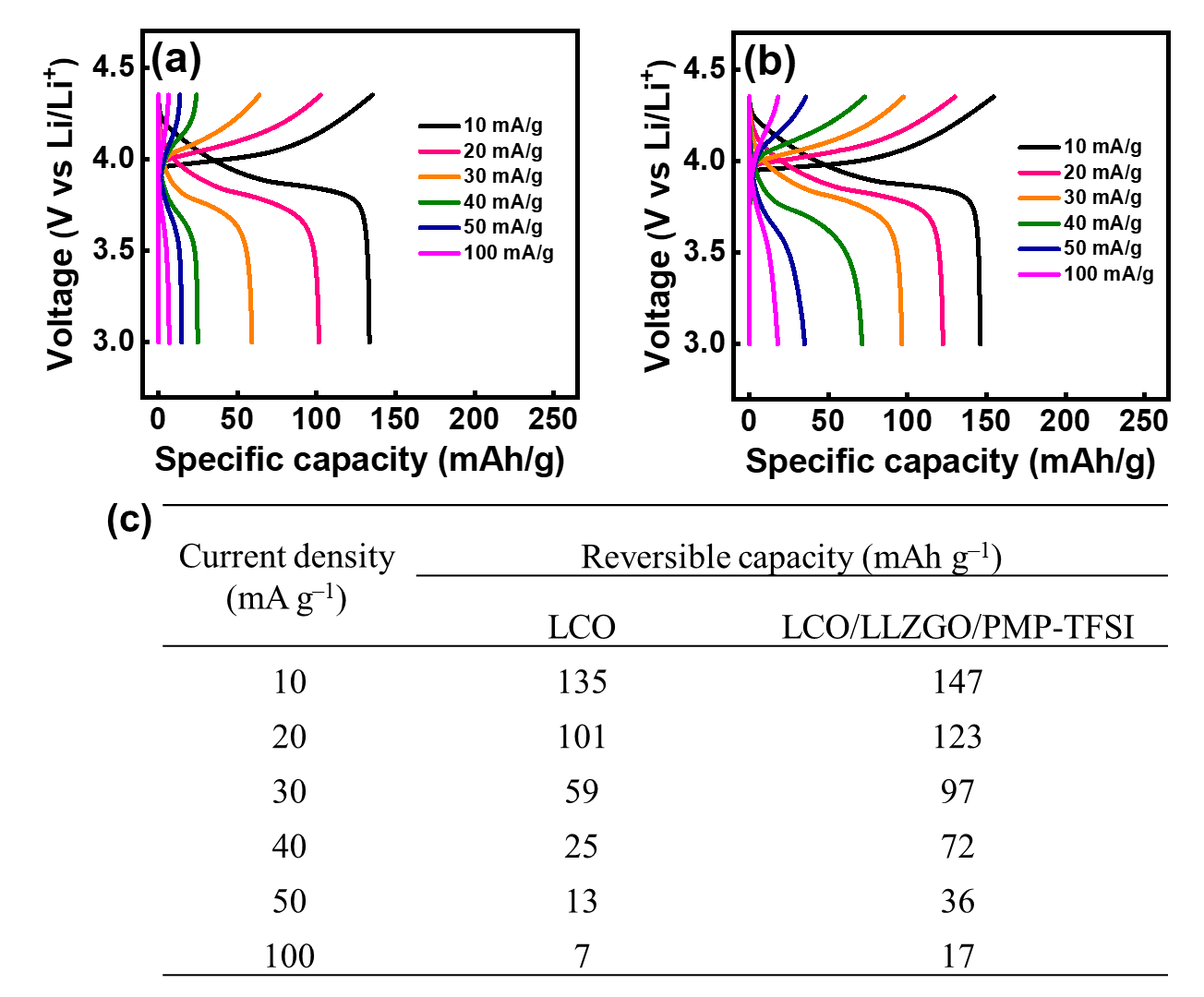


**Figure S13.** Galvanostatic charge-discharge curves of (a) LCO||Li cell and (b) LCO/LLZGO/PMP-TFSI||Li cell. (c) Comparison of reversible capacities of LCO measured at different rates in various cells.
